# Approaches for the design of reduced toxicant emission cigarettes

**DOI:** 10.1186/2193-1801-3-374

**Published:** 2014-07-22

**Authors:** David J Dittrich, Richard T Fieblekorn, Michael J Bevan, David Rushforth, James J Murphy, Madeleine Ashley, Kevin G McAdam, Chuan Liu, Christopher J Proctor

**Affiliations:** British American Tobacco, Group Research & Development, Regents Park Road, Millbrook, Southampton SO15 8TL UK

## Abstract

Cigarette smoking causes serious diseases through frequent and prolonged exposure to toxicants. Technologies are being developed to reduce smokers’ toxicant exposure, including filter adsorbents, tobacco treatments and substitutes. This study examined the effect of modifications to filter ventilation, variations in cigarette circumference and active charcoal filter length and loading, as well as combinations of these features in a reduced-toxicant prototype (RTP) cigarette, on the yields of toxicants in cigarette smoke. An air-dilution mechanism, called split-tipping, was developed in which a band of porous paper in the centre of the filter tipping functions to minimise the loss of effective filter ventilation that occurs at the high flow rates encountered during human-smoking, and to facilitate the diffusional loss of volatile toxicants. As compared with conventional filter ventilation cigarettes, split-tipping reduced tar and volatile smoke constituent emissions under high flow rate machine-smoking conditions, most notably for products with a 1-mg ISO tar yield. Furthermore, mouth level exposure (MLE) to tar and nicotine was reduced among smokers of 1-mg ISO tar cigarettes in comparison to smokers of cigarettes with traditional filter ventilation. For higher ISO tar level cigarettes, however, there were no significant reductions in MLE. Smaller cigarette circumferences reduced sidestream toxicant yields and modified the balance of mainstream smoke chemistry with reduced levels of aromatic amines and benzo[a]pyrene but increased yields of formaldehyde. Smaller circumference cigarettes also had lower mainstream yields of volatile toxicants. Longer cigarette filters containing increased levels of high-activity carbon (HAC) showed reduced machine-smoking yields of volatile toxicants: with up to 97% removal for some volatile toxicants at higher HAC loadings. Split-tipping was combined with optimal filter length and cigarette circumference in an RTP cigarette that gave significantly lower mainstream (up to ~90%) and sidestream (predominately 20%–60%) smoke yields of numerous toxicants as compared with a commercial comparator cigarette under machine-smoking conditions. Significantly lower mainstream and sidestream smoke toxicant yields were observed for an RTP cigarette comprising several toxicant reducing technologies; these observations warrant further evaluation in clinical studies where real-world relevance can be tested using biomarkers of exposure and physiological effect.

**Electronic supplementary material:**

The online version of this article (doi:10.1186/2193-1801-3-374) contains supplementary material, which is available to authorized users.

## Background

Epidemiological studies find that the health risks of cigarette smoking are dose-related and increase particularly with both duration of smoking and level of daily consumption (Doll et al. 1994). Cessation generally leads to a reduction in levels of tobacco-related relative risks to health, but the extent and speed of this reduction varies by disease (Doll et al. 1994; International Agency for Research on Cancer 2004). The health risks result from repeated and prolonged exposure to a range of tobacco smoke toxicants, of which more than 150 have been identified (Fowles and Dybing 2003; Green et al. 2007), in the diverse mixture of more than 6000 constituents that comprises cigarette smoke (Rodgman and Perfetti 2013). These toxicants are present in the mainstream smoke inhaled by a smoker and are also released between puffs into the atmosphere as constituents of sidestream smoke.

The concept of tobacco harm reduction, defined by the US Institute of Medicine (IOM) as "decreasing total morbidity and mortality, without completely eliminating tobacco and nicotine use" (Institute of Medicine 2001), is being considered by some regulators. One strategy described by the IOM is to reduce toxicant exposure in people who continue to use tobacco through what have been termed potential reduced-exposure products (PREPs) (Institute of Medicine 2001). PREPs were defined as products that substantially reduce exposure to one or more tobacco smoke toxicants as compared to exposures resulting from traditional tobacco products, and, that as a result, might be reasonably expected to reduce the risk of one or more specific diseases or other adverse health effects as compared to the risks associated with use of traditional tobacco products. In 2011, the IOM issued a further report (Institute of Medicine 2011) on the science needed to assess such products, which it identified as "modified-risk tobacco products" or MRTPs. The US FDA subsequently released draft guidelines on MRTPs (US Food and Drug Administration 2012) that lays a framework for future regulatory approval of such products. Both the IOM and FDA have cited the necessity of examining the effects of potential MRTPs on the health of individual tobacco users and the population as a whole to determine MRTPs’ possible contribution to tobacco harm reduction.

Various toxicological approaches have sought to identify which cigarette smoke toxicants make the greatest contribution to the various diseases caused by smoking (Fowles and Dybing 2003; US Food and Drug Administration 2012; Rodgman and Green 2003; Cunningham et al. 2011). There remains considerable uncertainty about both the identity of these toxicants and the extent of smoke toxicant reductions needed to decrease individual smokers’ relative risks of smoking-related diseases.

There have been numerous attempts over many years to develop cigarettes with reduced machine yields of toxicants, through use of measures such as modified agricultural and curing practices; selective removal of tobacco constituents; substitution of tobacco with alternative diluent materials; addition of chemical species to the tobacco blend; air dilution; and selective reduction of cigarette smoke toxicants by using filter materials such as cellulose acetate, resins and activated carbon; and the development of cigarettes that heat but do not burn tobacco. A number of technological approaches have been employed in commercial or test marketed cigarettes such as ADVANCE, OMNI, and Marlboro UltraSmooth (McAdam et al. 2012). Despite the vast range of approaches, however, none has successfully produced a cigarette with characteristics sufficient to qualify as a PREP or MRTP under the criteria established by the FDA and IOM.

We recently described four technological approaches to the reduction of toxicants in cigarette smoke, two of which modified the tobacco blend (McAdam et al. 2011; Liu et al. 2011a) and two of which modified the cigarette filter (Branton et al. 2011a, [b]). The two tobacco blend technologies—a tobacco substitute sheet (TSS) material and a tobacco blend treatment (BTT)—functioned to reduce the generation of toxicants at source within the burning cigarette. The two filter technologies—a high activity, polymer-derived, carbon adsorbent and an amine functionalised resin material (CR20)—functioned to remove volatile species from the smoke stream after formation. The toxicant classes targeted by these technologies are summarised in Table 1. A subsequent study described three reduced toxicant prototype (RTP) cigarettes with combinations of these blend and filter technologies that resulted smoking machine yield reductions of 10% to 95% for a range of tobacco smoke toxicants as compared to scientific controls and typical commercial products with equivalent ISO tar yields (McAdam et al. 2012). *In vitro* assessment studies indicated that the introduction of these technologies into experimental cigarettes (ECs) did not result in increased cytotoxic or genotoxic hazards (Combes et al. 2012, 2013; Fearon et al. 2012).

**Table 1:** **Toxicant reduction technologies and target toxicants**

Technology	Cigarette component	Description	Target toxicants
Tobacco substitute sheet (TSS)	Blend	Glycerol dilution technology	Whole smoke; additional selectivity volatile phenols
Blend treated tobacco (BTT)	Blend	Protease treated extracted tobacco	Phenolics and nitrogen-based constituents
High activity carbon (HAC)	Filter	Polymer-derived (synthetic), spherical carbon beads	Vapour-phase constituents
Amine functionalised resin	Filter	Amine group functionalised resin (CR20D)	Hydrogen cyanide, volatile acids, and carbonyls
Triethyl citrate	Filter	Filter tow plasticiser	Volatile phenols
Split-tipping (ST)	Filter	Ventilation technology; provides an extended ventilation zone	Gases and vapour-phase constituents; reduced overall exposure

A clinical trial of these RTPs, using a short-term (6-week) switching design, evaluated smoke toxicant exposure by measurement of various biomarkers of exposure (BoEs) among current smokers both before and after they switched from conventional cigarettes to the reduced toxicant prototype (RTP; ISRCTN 72157335) (Shepperd et al. 2013a). On average, the smokers who switched to RTPs with reduced machine yields of toxicants had reduced levels of corresponding BoEs. For vapour-phase toxicants such as acrolein and 1,3-butadiene, reductions of ≥70% were observed in both smoke chemistry and BoEs. Reductions in particulate phase toxicants such as tobacco-specific nitrosamines (TSNAs), aromatic amines and polyaromatic hydrocarbons (PAHs) depended on the technologies used, but in some cases were ≥80%, although some increases in other particulate phase toxicants were also observed.

The above studies indicate further research and development goals. First, reductions in mainstream smoke toxicants were focused on specific toxicant groups: for example, the RTPs containing TSS showed significant reductions in volatile toxicants but had little impact on aromatic amines, benzo[a]pyrene, and involatile phenols (hydroquinone, resorcinol and catechol), whereas the RTP containing BTT showed substantial reductions in most volatiles, and aromatic amines, but had little effect on phenols, isoprene and PAH. Thus, effectiveness across a wider range of toxicants may be advantageous for an RTP design. Second, reductions in sidestream emissions were also limited in scope: for example, RTP TSS1 did not show reduced sidestream benzo[a]pyrene, carbonyls, phenols and volatile hydrocarbons, whereas RTP BT1 showed few sidestream toxicant reductions other than TSNAs, HCN, pyridine and quinoline (McAdam et al. 2011). Although sidestream smoke is a secondary and less significant source of exposure to toxicants as compared with mainstream smoke, overall reductions in smokers exposure may be enhanced by cigarettes with reduced sidestream yields. The clinical trial also suggested opportunities for improving the RTPs as there were increases in BoE for nicotine, NNK, aromatic amines and PAHs among smokers of TSS1, and increases in BoE for nicotine and PAHs among smokers of BT1. Lastly, the acceptability and flavour quality of RTPs were rated by study subjects as suboptimal in comparison to commercial comparator cigarettes.

The aim of the present study was to broaden and extend the range of toxicant reductions, for both mainstream and sidestream smoke emissions. We describe the optimization of three additional approaches to toxicant reduction (split-tipping, smaller cigarette circumference, and increased carbon filter length and loading), their combination within a new RTP, and comparison between the performance of this cigarette and that of a relevant commercial product.

## Experimental

### Health Canada dataset

A Health Canada dataset from 2004 was used to assess the influence of cigarette weight and circumference on sidestream emission levels (Health Canada 2004). The dataset provided annually to Health Canada by cigarette manufacturers contains mainstream ISO and Health Canada Intense (HCI) data, and sidestream smoke data under ISO puffing parameters. Tar, nicotine and CO yields are available for 249 products on-sale on the Canadian market, and detailed smoke chemistry yields for 90 of those products. Prior to the present analysis on sidestream emissions duplicate values were removed from the dataset, as were the values for Vantage Max 15 KS and Vantage Rich 12 Ks both of which appeared to have incorrectly exchanged data for a number of analytes.

### Toxicant-reducing technologies

The ECs described in this study used several unconventional technological features designed to reduce target toxicants as detailed in Table 1.

### Tobacco substitute sheet (TSS)

The TSS was made from calcium carbonate, bound with sodium alginate, loaded with glycerol (approximately 12.5%) and coloured with caramel. When blended with tobacco and burnt in a cigarette, the TSS releases glycerol into smoke, thereby contributing to the total amount of nicotine-free dry particulate matter (NFDPM, or "tar"). As a result, it reduces the contribution of the tobacco combustion products (and their toxicants) to the overall NFDPM value; this process is termed "dilution" (McAdam et al. 2012).

### Blend-treated tobacco (BTT)

The tobacco was subjected to an aqueous extraction step; the extract was subsequently filtered to remove a proportion of polyphenols and proteins, and insoluble tobacco proteins were removed by treatment with protease. After washing and enzyme deactivation, the tobacco solids and aqueous extract were recombined. The BTT process results in reduced smoke yields of phenolics, aromatic amines, hydrogen cyanide and other nitrogenous smoke constituents; however, it also results in increased yields of formaldehyde and isoprene (Liu et al. 2011a).

### High-activity carbon (HAC)

The synthetic high-activity carbon used in the EC filters was obtained from Blucher GmbH (Mettmanner, Germany). The spherical beads possess a pore structure that differs from carbon typically used in commercial cigarettes with charcoal filters. As a result, this synthetic HAC has improved adsorption characteristics for a range of volatile smoke toxicants as compared with conventional carbon (Branton et al. 2011a).

### Amine-functionalised resin

An amine-functionalised resin in bead form (CR20, Diaion, Mitsubishi Chemical Corporation, Tokyo, Japan) was incorporated into the EC filters. CR20 offers selective reduction of HCN, volatile acids and carbonyls (Branton et al. 2011b).

### Triethyl citrate

Typically, commercial cellulose acetate cigarette filters are plasticised using triacetin (1,2,3-triacetoxypropane), at an inclusion level of approximately 6%–7% w/w, a process that enhances the ability of cellulose acetate to selectively reduce the yield of volatile phenolics in mainstream smoke (Norman 1999). In this study, the EC filters employed an alternative plasticiser, tri-ethyl citrate (1,2,3-triethyl 2-hydroxypropane-1,2,3-tricarboxylate), at approximately 16% w/w. Filters containing triethyl citrate demonstrate a greater reduction of phenols in mainstream smoke as compared with those containing triacetin (McAdam et al. 2012). The physical quality of the triethyl citrate plasticised filters was comparable to those of triacetin plasticised filters.

### Split-tipping

**Figure 1:**
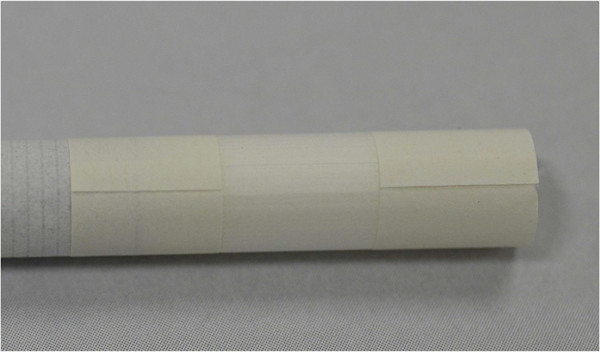
**Illustration of split-tipping as used in an experimental cigarette.**

Split-tipping filter technology can be used either as an alternative to conventional ventilation methods (such as on-machine laser [OML] ventilation) or as an additional means of filter ventilation. In conventional cigarettes, the filter is typically attached to the tobacco rod using a non-porous tipping wrapper; in filters incorporating split-tipping, however, the tipping paper is split into two separate strips. One part of the tipping wrapper covers the join of the filter to the tobacco rod, and the other part of the tipping wrapper is located at the mouth end of the cigarette. In between the separated tipping wrappers is a ‘split gap’, comprising an area of naturally porous paper (Figure 1). This ‘open’ gap section allows vapours and gases to diffuse in and out of the filter when the cigarette is being smoked. Diffusion of this kind does not occur using conventional filter ventilation systems in cigarettes.

Split-tipping may be particularly beneficial in products with short tobacco rods, where diffusion through cigarette paper is limited. However, we note that split-tipping technology is effective at influencing smoke yields only under machine-smoking regimes, or under human-smoking conditions, where the filter ventilation zone is not blocked. This means that split-tipping is not effective with the Health Canada Intense machine-smoking regime (see below), where all means relating to filter ventilation are fully closed.

The ECs described in this study incorporated both split-tipping plus OML filter ventilation to obtain target NFDPM yields.

### Cigarette specifications

#### Experimental cigarettes

All of the ECs used to examine the characteristics of split-tipping, cigarette circumference and carbon filter-adsorbent loading had a cigarette length of 83 mm, and filters constructed mainly from cellulose acetate fibres. A number of the filters used in this study contained filter adsorbents interspersed randomly amongst the cellulose acetate fibres in an arrangement known as a "Dalmatian" format.

The ECs designed to investigate split-tipping characteristics had a tobacco rod/filter length of 56 mm/27 mm, a circumference of 24.6 mm, and US style tobacco blends: one tobacco blend was used for the 1 and 4 mg ISO tar yield cigarettes; a second tobacco blend was used for the 7 and 10 mg ISO tar yield cigarettes. The filter was plasticised with triacetin. Papers with a range of air permeabilities were used for the porous area of the split-tipping (Additional file 1: Table S1).

The ECs used to examine the influence of circumference on smoke yields had a circumference of 17.0, 21.0 or 24.6 mm; a fixed tobacco blend composition (flue-cured Virginia blend modified with 20% TSS), density and rod firmness; and a 27 mm long single stage ("mono") cellulose acetate filter plasticised with 7% triacetin (Additional file 1: Table S2).

The ECs used to examine the effect of higher HAC loadings had a circumference of 17, 21 or 24.6 mm; a tobacco rod/filter length of 56 mm/27 mm, 50 mm/33 mm or 46 mm/37 mm; and a fixed tobacco blend composition (flue-cured Virginia blend modified with 20% TSS). The filters had a two-stage (dual) construction with a 15 mm cellulose acetate mouth-end section and a variable 12, 18 or 22 mm tobacco-end section containing HAC; triethyl citrate was used as the plasticiser (Additional file 1: Table S3). HAC was incorporated in a Dalmatian format at various loadings of up to 88 mg. The ECs were designed to a target ISO tar yield of 7 mg. Cigarettes with a circumference of 24.6 mm were manufactured with carbon loadings of 48, 72 and 88 mg in filters of 27, 33 and 37 mm length using a fixed adsorbent packing density of 4 mg of HAC per mm of bed length. For the 21 mm circumference cigarette, carbon loadings of 30, 45 and 55 mg were created in 27, 33 and 37 mm filters, respectively, with an adsorbent packing density of 2.5 mg/mm. For the 17 mm circumference cigarettes, only filter lengths of 27 and 33 mm length (with corresponding carbon loadings of 20.4 and 30.6 mg at an adsorbent packing density of 1.7 mg/mm) could be manufactured successfully at a 7 mg ISO tar yield. The reduced tobacco rod length (and therefore tobacco weight) has lower tar potential, and changes to the cigarette paper were also necessary to meet the target of 7 mg ISO tar. Note that tri-ethyl citrate was used as the plasticiser in all cigarettes except for the control product for the 21 mm series, wherein a suitable reference cigarette existed with triacetin as a plasticiser; in addition, the split-tipping gap length increased with increasing filter length. It was considered that these two factors were unlikely to significantly influence the yields of the toxicants examined.

#### Reduced-toxicant prototype and comparator cigarettes

The RTP cigarette ("RTP2") had a circumference of 21 mm and a length of 83 mm (Additional file 1: Table S4). The blend combined low toxicant precursor tobaccos (26.5% flue-cured and 8.5% oriental tobaccos), BTT (50%) and TSS (15%). A cigarette paper of 50 Coresta units (the volume of air passing through 1 cm^-2^ per minute at a constant pressure difference of 1.0kPa) wrapped the tobacco rod. A three-stage (triple) cellulose acetate filter was used with a 15-mm mouth-end a 10-mm central section containing 20 mg of CR20 resin (distributed among the cellulose acetate fibres in a Dalmatian format), and a 12-mm section containing 50 mg of HAC in Dalmatian format at the tobacco-rod end of the cigarette. A white tipping paper was used with a 10-mm split-tipping gap and OML, leading to an overall ventilation of 35%.

The commercial comparator product ("CC7") was a British American Tobacco manufactured product of 83 mm in length and 24.6 mm in circumference (Additional file 1: Table S4). It was based on the brand Lucky Strike Silver on sale in Germany in 2012 and contained a US-blended style of tobacco and a 27-mm single-stage cellulose acetate filter wrapped with two tipping paper variants for switching purposes in a subsequent clinical study, a white tipping paper variant and a white/cork tipping variant. The comparator cigarettes were manufactured in a non-branded form but were alpha-numerically coded.

### Analytical methods

#### Smoking machine regimes

Overall, six smoking regimes were used in the present study to measure mainstream smoke yields. Two smoking regimes in active regulatory use were employed: namely, ISO 4387 ( 2000), for ISO smoking conditions (a 35-ml puff of 2 seconds duration taken every 60 seconds with ventilation unobstructed; abbreviated as 35/2/60 VO); and ISO 3308 ( 2000) for NFDPM and nicotine analysis. The Health Canada Intense (HCI) smoking regime of 55/2/30 VC (ventilation closed) was also used. In addition, three regimes were used to provide greater analytical sensitivity and insight into behaviour with partial ventilation blocking: namely, the regime of Siu et al. ( 2012), which is effectively the HCI smoking regime but without filter ventilation blocking (55/2/30 VO; abbreviated as HCI-VO); the Option B intense smoking regime proposed by ISO TC126 Working Group 9 (60/2/30 V_50%_ with ventilation 50% blocked; abbreviated as WG9); and the same smoking conditions with ventilation open (60/2/30 VO). The specific smoking regimes used to measure mainstream yields in the studies described in this paper are detailed in each of the subsections of the Results section. For sidestream smoke analysis the ISO puffing parameters were used, as emission levels are generally higher than found with other smoking regimes.

#### Smoke analysis

Two different laboratories were used to determine toxicant yields in smoke owing to logistical issues associated with laboratory moves at the time of analysis. Mainstream smoke analyses for examining the influence of circumference and adsorbent loading on toxicant yields were conducted by British American Tobacco (analytical methods are available http://www.bat-science.com). Comparisons of the mainstream and sidestream toxicant yields of the RTP (RTP2) cigarette with the commercial comparator cigarette (CC7) were conducted by Labstat International ULC (Kitchener, Canada).

Smoke constituent measurements characterising the performance of split-tipping were also conducted by British American Tobacco other than levels of smoke metals, which were analysed by Labstat International ULC (Kitchener, Canada). Additional file 1: Table S5 lists the method numbers and reporting limits for all of the analyses used. Prior to smoke chemistry analysis, cigarettes were conditioned according to the specifications of ISO 3402 ( 1999).

#### Tobacco blend analysis

Tobacco blends used in the ECs described in this work were analysed for nicotine, sugars, total and protein nitrogen, polyphenols, metals, TSNAs, and benzo[a]pyrene (Additional file 1: Table S6). The analyses were conducted by British American Tobacco ( British American Tobacco website). Reporting limits for these analyses are given in Additional file 1: Table S5.

#### Mouth-level exposure studies

Ventilation-flow relationships were measured on unlit cigarettes using a bespoke experimental setup. A vacuum pump flow-generating unit was connected to a Furness Controls FCO96E-20L laminar Flow Element connected to Furness Controls FCO510 micromanometers.

#### Mouth-level exposure studies

Study fieldwork comparing human exposure from split-tipping and conventionally ventilated cigarettes was conducted by a market research agency in Hamburg, Germany, between September and November 2012. The study design involved 50–60 smokers for each cigarette category of 1 mg, 4 mg, 7 mg and 10 mg ISO tar. The subjects smoked at least eight cigarettes per day, had been smoking their usual product for 6 months or longer, and were aged between 21 and 64 years. Pregnant females were excluded from the study groups. The gender and age split of each group were representative of the demographics of the tar bands in Germany. Subjects who complied with the screening criteria were briefed on the study protocol and gave written informed consent to participate in the study.

Each subject smoked the test EC (split-tipping) for 5 days or a control cigarette of their usual ISO tar for 5 days. The subjects smoked the supplied cigarettes in their usual manner and environment, starting on the day of the visit to a central location for cigarette distribution, and continuing for a further 5 days before switching to the second product for the same time period. Each subject was provided with enough cigarettes to smoke for 5 days, on the basis of their self-reported average daily cigarette consumption declared at the start of the study, plus an additional 30% rounded up to the nearest pack. The first full day of smoking after the central location visit was considered to be day 1. Subjects were given a filter collector/cutter designed to cut a 10-mm portion from the mouth-end filter section and were requested to use it to cut and collect 20–25 part-filters from the study cigarettes smoked on days two, three and four. Subjects recorded the number of study cigarettes that they smoked each day using a consumption diary. They were also instructed to record any non-study cigarettes that they smoked during the study. The subjects returned to the central location on day 5 with their filter collector/cutter containing cut spent filters, and the completed consumption diary. The subjects were supplied with the next study product, a new filter collector/cutter and a consumption diary, and were instructed to repeat the activities conducted with the first study cigarette. The subjects returned to the central location for a third visit to complete the study.

Smokers’ MLE was estimated by a previously described filter analysis methodology (St Charles et al. 2009). In brief, smoke toxicant yields among smokers were estimated by the relationship between the amount of tar and nicotine retained within the filter and the amount passing through the filter to the smoker, as defined by calibration smoking using a smoking machine. Calibrations were established by machine smoking each product over a wide range of typical human smoking behaviour parameters (e.g. puff volumes, durations and flows). Each smoker’s spent filters collected in the filter cutters were split randomly into three groups of five tips, which were analysed independently on different days. The length of each filter tip was measured (±0.1 mm), and recorded before being extracted in methanol (containing n-heptadecane as an internal standard). The extracts were analysed for both tip nicotine using gas chromatography and tar using UV absorbance with a variable wavelength detector set at 310 nm as described previously (St Charles et al. 2009).

MLE to nicotine was obtained for each extract by using the measured human tip nicotine values and the linear regression equation obtained by plotting mainstream smoke nicotine yield versus tip nicotine data obtained during calibration. Similarly, MLE to tar was obtained using UV absorbance per tip data and the linear regression equation derived by plotting mainstream smoke NFDPM yield versus UV absorbance per tip during calibration.

### Statistical analysis

Tobacco blends and smoke yields were statistically compared between different cigarette types by two-tailed, unpaired, Student’s t-tests or ANOVA analysis performed using Minitab v16 (Minitab Ltd, Coventry, UK). A *P* value of >0.05 was considered to be non-significant.

In experiments examining smokers’ MLE to Tar and Nicotine ANOVA GLM, with subject as a random factor, was used to compare smokers’ MLE to tar and nicotine obtained for the control and split tipping products within each ISO pack tar group. Significant differences were set at the 5% level (p < 0.05).

## Results

### Split-tipping ventilation system

#### Reduced ventilation dependency on flow rate

Cigarette filter ventilation is used to dilute, during a puff, mainstream smoke generated in the tobacco burning zone with air entering through holes or perforations in the filter tipping paper, thereby reducing mainstream smoke yields (Norman 1974). Many cigarettes have a ventilation system in which both the tipping paper and a non-porous plug wrap are perforated by an ‘on-machine laser’ (OML). Ventilation levels are measured on unlit cigarettes as the percentage of airflow entering the ventilation holes at a puff-flow rate of 17.5 mL/s (equivalent to ISO smoking conditions).

Filter ventilation shows flow rate dependency, typically decreasing when the flow rate through the filter increases (Lewis and Norman 1986; Mathis 1987). Puff flow rates produced by smokers tend to be higher than the 17.5 mL/s used to measure ventilation levels. For example, the US Surgeon General (Office of the Surgeon General 1988) reported a range of peak puff flow rates of 28 to 48 mL/s on the basis of data obtained from 5 studies (Gritz et al. 1983; Guillerm and Radziszewski; Nil et al. 1984, 1986; Medici et al. 1985). More recently, Hammond et al. (Hammond et al. 2006) reported a mean ‘average puff flow rate’ of 38.6 mL/s from 51 smokers of 9–15-mg ISO tar yield Canadian cigarettes. As compared with the 17.5 mL/s average flow rate of the ISO smoking regime, the higher flow rates produced by smokers would reduce both the actual filter ventilation and filter efficiency and thereby increase the smoke yield from the cigarette. This physical phenomenon of ventilated cigarettes has been described as ‘elasticity’ by some researchers (Chaiton et al. 2005; Hammond et al. 2005).

In the present study, split-tipping—a novel method of filter ventilation aimed at reducing the flow rate dependency of filter ventilation (see Experimental and Figure 1)—was tested against standard cigarettes with OML ventilation. Four matched pairs of king-sized cigarettes were prepared with ISO tar yields of 1, 4, 7 and 10 mg. The cigarettes in each pair were prepared with either traditional OML, or split-tipping (10-mm split gap) in combination with OML ventilation to achieve target tar yields, but otherwise had essentially identical composition (Additional file 1: Table S1).

**Figure 2:**
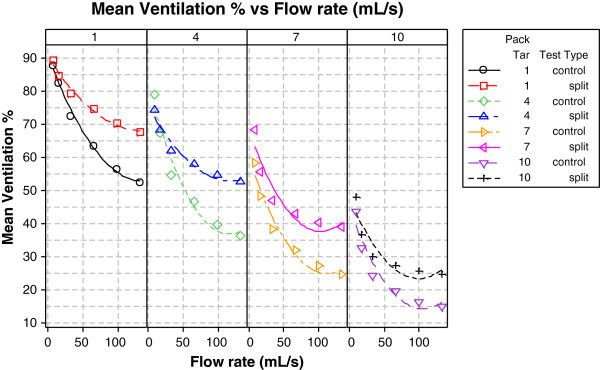
**Role of split-tipping in diminishing the impact of flow-rate on ventilation efficiency.**

The effectiveness of split-tipping in minimising the reduction in effective ventilation with increasing flow rate is shown in Figure 2 for unlit cigarettes. Within each ISO tar level, the effective ventilation decreased as the flow rate increased for both cigarette types. As the flow rate increased, however, higher ventilation levels were measured for the split-tipping EC than for the OML control at all measured flow rates. The difference in effective ventilation increased with flow rate, and was also more significant under the higher ventilation conditions present in low ISO tar cigarettes. Thus, split-tipping reduced the ventilation dependency on flow rate in unlit ECs under laboratory conditions. These findings suggest that the split-tipping ventilation system might reduce toxicant exposure from cigarettes under human smoking conditions.

#### Reduced toxicant yields in mainstream smoke

The effect of split-tipping on toxicant yields in mainstream smoke was measured for both volatile and condensed phase toxicants under two machine smoking regimes (ISO, and a higher flow rate 60/2/30 VO smoking regime). The smoke toxicants quantified were a combination of toxicants of active regulatory interest and precursors of available biomarkers of exposure (Table 2) (McAdam et al. 2012). For each smoking regime, comparisons were conducted between the split-tipping and control products within an ISO tar band. For 1-mg ISO tar products, the split-tipping tar yield was lower than the OML cigarette tar yield; the reduction in tar yield was larger (32%) at the higher flow rate than at the ISO flow rate (17%, not significantly different at 95% confidence level). Thus, the split-tipping mechanism maintained more effective filter ventilation at elevated flow rates.

**Table 2:** **Effect of split-tipping ventilation on mainstream smoke yields of toxicants**

Smoke constituent	ISO smoking regime								60/2/30 VO smoking regime							
	1 mg ISO tar		4 mg ISO tar		7 mg ISO tar		10 mg ISO tar		1 mg ISO tar		4 mg ISO tar		7 mg ISO tar		10 mg ISO tar	
	Control	Split	Control	Split	Control	Split	Control	Split	Control	Split	Control	Split	Control	Split	Control	Split
1-Aminonaphthalene (ng/cig)	2.3	1.6	5.1	5	8.3	8.3	9.7	10.8	9.8	7.4	15	17	20.2	21.4	24.2	22.3
2-Aminonaphthalene (ng/cig)	2	1.4	5	4.4	8.5^ǂ^	7.6^ǂ^	10.2	10.6	9.4^ǂ^	6.8^ǂ^	14.4	14.3	18.8	21.5	22	21.7
3-Aminobiphenyl (ng/cig)	0.5^ǂ^	0.3^ǂ^	1.3	1.1	2.2^ǂ^	1.9^ǂ^	2.8	2.7	2.2	1.8	3.5	3.4	4.9	5.2	5.6	6.2
4-Aminobiphenyl (ng/cig)	0.3	0.2	0.8	0.8	1.4	1.4	1.8	1.7	1.6	1.4	2.5	2.4	3.4	3.7	3.9	4.1
Ammonia (μg/cig)	1	<RL	3.3	4	6.1^ǂ^	6.9^ǂ^	9.4	10.1	4.6^ǂ^	3.3^ǂ^	9.5^ǂ^	7.5^ǂ^	13	11.4	16	15.3
Benzo(a)pyrene (ng/cig)	2.2	1.4	6.8^ǂ^	4.2^ǂ^	7.8	7.7	10.4	9.2	6.2^ǂ^	4.8^ǂ^	10.3	10.4	16.6	17	19.3	18.3
Acetaldehyde (μg/cig)	78	54.8	215.3	170.4	352.3	314.7	496.6	455.1	402.2^ǂ^	249.6^ǂ^	720.4^ǂ^	553.1^ǂ^	905	798	1037.2	896.2
Acetone (μg/cig)	44.9	32.4	121.5	97.8	201.2	183.2	273.5^ǂ^	252.1^ǂ^	242^ǂ^	152^ǂ^	410.6^ǂ^	317.8^ǂ^	507.9	446.3	570.2	494.1
Acrolein (μg/cig)	5.8	3.8	19.6	14.9	35.9	30.1	52.2	47.8	40.6^ǂ^	21.9^ǂ^	86^ǂ^	61.2^ǂ^	110.7	94.4	132.1	111.1
Butyraldehyde (μg/cig)	6.7	5.3	15.7	13	25.4	23.2	35.5	32.3	34	22.9	57.2	44.3	72.6	64.1	83.1	71.9
Crotonaldehyde (μg/cig)	1.3	0.8	4.7	3.3	10.2	7.6	17	14.1	15.1	9.1	27.7	19.7	36.5	31.8	44.7	37.8
Formaldehyde (μg/cig)	1.3	0.9	5.9	4.9	13.5	11.9	24.7	22.9	9.3	6	23.7^ǂ^	16.1^ǂ^	41.4	34.3	64.8	50.7
Methyl ethyl ketone (μg/cig)	10.8	7.9	29	23.2	50.9	45.5	70.1	64.9	68.8	42.1	108.9	86.7	140.4	125.4	153.9	138.1
Propionaldehyde (μg/cig)	7.9	5.7	20.9	16.9	34.3	31.1	47.2	43.7	39.2^ǂ^	24.8^ǂ^	68.2^ǂ^	54.2^ǂ^	86.8	78.1	98.7	87.4
Hydrogen cyanide (μg/cig)	8	6	33.9^ǂ^	23.1^ǂ^	62.2	51.2	95.2	80.5	100.1^ǂ^	49.6^ǂ^	233.1^ǂ^	141.3^ǂ^	291.2	230.7	369.1	288.2
CO (mg/cig)	1.2	1	4.4^ǂ^	3.4^ǂ^	6.6	6.2	9.7^ǂ^	8.7^ǂ^	8.7^ǂ^	5.2^ǂ^	15.6^ǂ^	12.3^ǂ^	18.2	17	23^ǂ^	20.5^ǂ^
NO (μg/cig)	43.4^ǂ^	70.7^ǂ^	97.1^ǂ^	84.5^ǂ^	137	129.6	175.6	167.4	185.7^ǂ^	142.9^ǂ^	297.5	252.5	347.9	329	371.6	383.5
Catechol (μg/cig)	7.7	6.1	23.9	23.8	38.3	38.1	50.4	48.7	39.2	37.4	71.6	70.5	106.3	105.8	129	129.8
Hydroquinone (μg/cig)	6.7^ǂ^	4.9^ǂ^	24.3	23.2	39.9	39.3	50.1	48.6	40.9	36.3	73.9	71.7	112.1	104	133.6	127.7
M-cresol (μg/cig)	<RL	<RL	1.2	1.3	2.3	2.5	3.3	3.5	2.2	2.4	4	4.8	5.3	6.4	5.8	7.2
O-cresol (μg/cig)	<RL	<RL	1.4	1.6	2.9	3.1	4.2	4.5	2.5	2.9	4.9	6	6.6	8.2	7	9.1
P-cresol (μg/cig)	0.5	0.5	2.9	3.2	5.7	6.1	8.5	8.7	5.4	5.7	10.4	12.4	13.8	17	15.2	18.7
Phenol (μg/cig)	<RL	<RL	4.4	5.2	9.7	10.7	15.2	16.1	8.4	10.2	17.5	22.6	23.4	29.7	25.1	33.3
Resorcinol (μg/cig)	<RL	<RL	0.6	0.5	0.9	0.8	1.2	1.1	0.9	0.8	1.5	1.5	2.4	2.3	3	2.8
Pyridine (μg/cig)	<RL	<RL	2.1^ǂ^	1.5^ǂ^	5	4	8	6.4	4.2^ǂ^	2.1^ǂ^	11.9^ǂ^	8.4^ǂ^	19.2^ǂ^	16.3^ǂ^	26.8	24.1
Quinoline (μg/cig)	<RL	<RL	0.2	0.2	0.4	0.3	0.5	0.4	0.3^ǂ^	0.2^ǂ^	0.5	0.4	0.7	0.6	0.7	0.7
Styrene (μg/cig)	1	<RL	3.1^ǂ^	1.7^ǂ^	5.9	4	8.4	6.4	7^ǂ^	3^ǂ^	14.3^ǂ^	9.4^ǂ^	18^ǂ^	15.1^ǂ^	21.3	19.8
N-Nitrosoanabasine (NAB) (ng/cig)	1.6	1.3	4.4	4.6	5.8	5.9	7.8	8.3	6.1	5.3	12.3	10.7	13.3	14.2	16.7	16
N-Nitrosoanatabine (NAT) (ng/cig)	13.9	10.8	36.3	36.3	48.6	51.1	68.9	71.1	51	43.8	98.1	86.9	119.6	120.8	153.6	144.5
N-Nitrosonornicotine (NNN) (ng/cig)	18.3	14.8	49.7	46.3	61.7	64.6	90.6	99	70.3	59.3	139.3^ǂ^	112.3^ǂ^	155.2	158.1	195.5	184.9
N-Nitrosonornicotine ketone (NNK) (ng/cig)	7.3	5.7	19.4	18.5	27.4	30.1	40.2	50.2	25.8	18.6	49.2	42	67.5	70.4	80.5	78.9
1,3-Butadiene (μg/cig)	6.7^ǂ^	4.6^ǂ^	22^ǂ^	17^ǂ^	32	28.2	42.6	39	40.4^ǂ^	11.3^ǂ^	67.6	57.1	82	78.3	96.6	92
Acrylonitrile (μg/cig)	1.1	0.7	4.6^ǂ^	3.1^ǂ^	7.7	6.1	11.2	9.4	10.5^ǂ^	2.4^ǂ^	18^ǂ^	13.8^ǂ^	24.1^ǂ^	21.1^ǂ^	28.5	25.7
Benzene (μg/cig)	8^ǂ^	5.7^ǂ^	25.4^ǂ^	19.3^ǂ^	37	33.3	47.8	42.5	45.5^ǂ^	13.8^ǂ^	73.6	60.2	87	83.6	100	92.5
Isoprene (μg/cig)	69.6	53.9	201^ǂ^	158.9^ǂ^	299	259.1	388.3	357.2	369.2^ǂ^	114.9^ǂ^	579.4	509.3	718.7	698.4	836.6	811
Toluene (μg/cig)	11.6^ǂ^	6.7^ǂ^	41.3^ǂ^	28.3^ǂ^	63.9^ǂ^	52.8^ǂ^	83.4^ǂ^	68.2^ǂ^	86.6^ǂ^	21.9^ǂ^	124.8	103.3	152.9	150	170.9	166.9
Arsenic (ng/cig)	BDL	BDL	BDL	BDL	NQ	NQ	NQ	NQ	BDL	BDL	NQ	NQ	NQ	NQ	NQ	NQ
Cadmium (ng/cig)	NQ	NQ	6.5	5	11.3	10.1	20.6^ǂ^	15.5^ǂ^	12.2^ǂ^	6.4^ǂ^	30.1	23.3	39.2	33.9	51.8	48
Chromium (ng/cig)	BDL	BDL	NQ	NQ	NQ	NQ	NQ	NQ	BDL	BDL	NQ	BDL	NQ	NQ	NQ	NQ
Mercury (ng/cig)	NQ	NQ	NQ	NQ	1.7	1.5	2.2	2	NQ	NQ	4.3	3.9	4.5	4.2	4.8	4.8
Nickel (ng/cig)	NQ	NQ	NQ	NQ	NQ	NQ	NQ	NQ	BDL	BDL	BDL	BDL	BDL	BDL	BDL	BDL
Lead (ng/cig)	NQ	NQ	NQ	NQ	NQ	NQ	NQ	NQ	BDL	BDL	NQ	NQ	NQ	NQ	NQ	NQ
Selenium (ng/cig)	BDL	BDL	BDL	BDL	BDL	BDL	BDL	BDL	BDL	BDL	BDL	BDL	BDL	BDL	BDL	BDL
Tar	0.6	0.5	3.4	3	6.4	6.3	9.8	9.2	**5.6** ^ǂ^	**3.8** ^ǂ^	12.6	11.3	18.4	18.5	25.4	23.5
Nicotine	< 0.1	< 0.1	0.32	0.3	0.58	0.55	0.79	0.78	**0.51** ^ǂ^	**0.36** ^ǂ^	1.07	0.99	1.46	1.46	1.84	1.77

^ǂ^Indicates that the difference in yield between the split-tipping and control products was significant at a 95% confidence level.

Several other toxicants showed reduced yields from the 1-mg ISO tar split-tipping EC in comparison to the OML cigarette under the high flow-rate smoking regime. Some particulate compounds (aromatic amines, benzo[*a*]pyrene) were reduced at a level comparable to that of tar under the high flow rate smoking regime, implying that the operating mechanism might be a general reduction in the overall quantity of smoke generated by the cigarettes. Data for NO were somewhat inconsistent, showing contradictory behaviour with the 1 mg ISO product. Other gaseous and volatile compounds (including volatile hydrocarbons and small aromatics, pyridine, cadmium, CO, HCN and some carbonyls) were reduced by more than the tar value (*P* < 0.05) under the high flow rate smoking regime; the greatest reduction was observed for volatile hydrocarbons. These observations are consistent with diffusional losses of volatile toxicants through the cigarette wrapper or the split-tipping porous paper gap in the filter.

Similar, but fewer, compounds were reduced more than tar for the 4-mg ISO tar split-tipping EC as compared with the control product. For the 7-mg and 10-mg ISO tar yield ECs, however, few compounds were significantly reduced, showing that the effect of split-tipping was diminished at low ventilation levels.

Under the lower flow rate ISO smoking regime, mainly volatile species showed reduced yields from the split-tipping EC as compared with the control cigarette. For the 1-mg EC, however, fewer compounds were significantly reduced as compared with the higher flow rate regime (60/2/30 VO). By contrast, comparable numbers of toxicant reductions were observed for the higher ISO tar yield cigarettes across the two smoking regimes. Taken together, these results indicate that split-tipping is less effective under slow puffing flow rates, consistent with its anticipated mode of operation.

We note that small increases in the yield of cresols and phenol were observed for the split-tipping ECs. Although not statistically significant, these increases were observed consistently for all four ISO tar ECs under both smoking regimes, implying a consistent trend in the behaviour of these species. This may reflect differences in the interaction of the volatile phenolics with cellulose acetate, owing to the pathway of smoke in the filter and the width of the split-tipping ventilation zone.

#### Reduced tar and nicotine yields among smokers

The effect of split-tipping on exposure to nicotine and tar among smokers was examined by MLE studies. There was no significant difference in cigarette consumption between the groups smoking the split-tipping products and those smoking the control product.

Among smokers of 1-mg ISO tar cigarettes, those smoking split-tipping ECs had significantly lower MLE to tar and nicotine (4.1 mg/cig and 0.31 mg/cig, respectively) than those smoking control cigarettes (5.1 mg/cig and 0.40 mg/cig, respectively; *P* < 0.001). Among 7-mg ISO tar smokers, slightly lower (5%) MLE to nicotine was found for the control product compared as with the split-tipping product (*P* = 0.039). No significant differences between the products were found for smokers of 4-mg and 10-mg ISO tar cigarettes. Taken together, these data suggest that the split-tipping technology in King-sized format might be an effective mechanism for reducing exposure to certain toxicants among smokers, but only for cigarettes with very low ISO tar yield where ventilation levels are greatest.

### Influence of cigarette circumference on smoke yields

The tobacco weight used in a cigarette has an important role in determining both the level of smoke emission from a cigarette and product quality (Health Canada 2004). For example, the importance of tobacco weight in determining mainstream tar and nicotine yields has been demonstrated (Yamamoto et al. 1984) and is likely a determinant of sidestream emissions, although other features of cigarette design also influence emission levels and product quality (Health Canada 2004).

**Figure 3:**
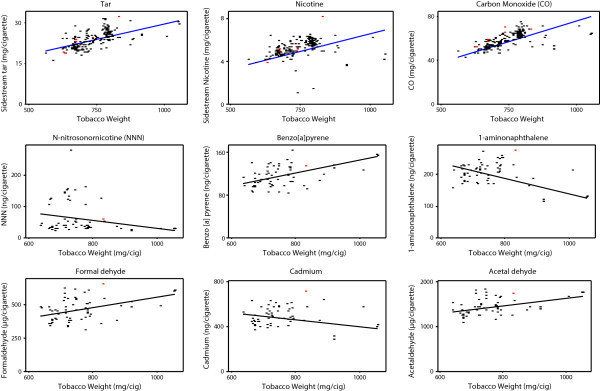
**Influence of cigarette tobacco weight on ISO sidestream yields of toxicants from Canadian cigarettes (Health Canada** 2004 **).**

For a fixed length cigarette, tobacco mass can be reduced by decreasing either the tobacco packing density or the circumference. In addition to influencing smoke yields, however, significantly reducing the tobacco packing density can affect the integrity of the tobacco rod, puff number and pressure drop (Health Canada 2004), each of which can affect product quality and acceptability among consumers. We first examined the influence of tobacco weight on sidestream smoke yields using data submitted to Health Canada in 2004 (Health Canada 2004). Figure 3 shows that sidestream toxicant yields increase with increasing tobacco mass for some analytes (tar, nicotine, CO, 2-aminonaphthalene, 4-aminobiphenyl, benzo[a]pyrene, all measured carbonyls, cresols and isoprene); however, the sidestream yields of other analytes were found to decrease with increasing tobacco mass (e.g. TSNAs, HCN, 1-aminonaphthalene, 3-aminobiphenyl, NO, and a number of volatile species). Thus, although tobacco weight influences the yield of sidestream smoke from cigarettes, other factors strongly contribute to individual toxicant yields. The cigarettes in the Health Canada dataset represent various tobacco blends (although the great majority, but not all, of the blends will be flue-cured in nature) and cigarette constructions, for which little information is available. The impact on this is most apparent for species such as TSNAs, whose yields in smoke are highly dependent on their levels in the tobacco blend (Baker 1999).

The Health Canada data also suggest that cigarette circumference has little effect on smoke yields over and above changes in tobacco weight (Figure 3). To clarify the effectiveness of tobacco weight reduction through decreased circumference on yields of individual sidestream smoke constituents, three 1-mg ISO tar yield ECs (K111, D111 and S111) were manufactured that differed in circumference, but maintained the same 1-mg ISO tar yield, tobacco blend and cigarette design (other than changes in filter pressure drop and paper permeability necessary to achieve the same ISO tar yield across the three cigarettes; Additional file 1: Tables S2 and S6).

**Figure 4:**
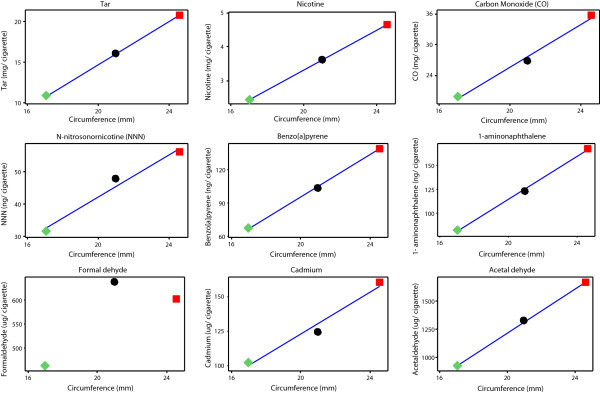
**ISO sidestream yields from 1-mg ISO tar cigarettes with circumferences between 17.0 and 24.6 mm.**

The ISO sidestream yields from these products are shown in Table 3 and in Figure 4. The data show a progressive reduction in sidestream yields for all constituents other than formaldehyde as the cigarette circumference (and tobacco weight) are reduced. The formaldehyde yields rise from 24.6 to 21-mm circumference before falling to their lowest level from the 17-mm circumference product. These data show that reducing tobacco weight in a cigarette is an effective mechanism for reducing sidestream smoke toxicant emissions, and circumference reduction is one route to achieve this.

**Table 3:** **Effect of changing circumference on mainstream and sidestream smoke yields of toxicants**

	Sidestream smoke			Mainstream smoke (HCI-VO smoking regime)					
Smoke constituent	(ISO puffing conditions)								
	K111 24.6 mm	D111 21.0 mm	S111 17.0 mm	K711 24.6 mm	D711 21.0 mm	S711 17.0 mm	K111 24.6 mm	D111 21.0 mm	S111 17.0 mm
Ammonia (μg/cig)	4923	3502	2263	18.7	15.6^*^	16.2^*^	6.1	4.2^*^	4.3^*^
1-Aminonaphthalene (ng/cig)	167	123	83.4	12.2^*^	11.7^*^	6.9	7.4	5.7	4.3
2-Aminonaphthalene (ng/cig)	148	110	81.6	10.3	8.4	5	6.7	3.7	2.6
3-Aminobiphenyl (ng/cig)	32.2	24.7	17.1	2.3	1.8	1.3	1.3	0.9	0.7
4-Aminobiphenyl (ng/cig)	20.5	16.6	11.2	1.9	1.5	1.2	1.1	0.8	0.7
Benzo[a]pyrene (ng/cig)	139	103	67.1	18.6	15.5	8.4	9.6	7.4	5.8
Hydrogen cyanide (μg/cig)	73.6	65.0	52.0	176.9	148.7^*^	147.4^*^	46.6^*^	24.5	42.8^*^
Acetaldehyde (μg/cig)	1663	1320	925	636.5	522.9^*^	486.9^*^	284.5	149^*^	163.3^*^
Acetone (μg/cig)	726	565	389	323	251.8	221.9	144.6	72.8^*^	80.6^*^
Acrolein (μg/cig)	382	333	238	94.4	89	80.7	38.2	20.3^*^	26.2^*^
Butyraldehyde (μg/cig)	101	78.9	57.4	48.7	39.3^*^	37^*^	20.9	9.9	13
Crotonaldehyde (μg/cig)	85.2	71.3	51.3	28.7	22.5^*^	23.6^*^	8.3^*^	2.9	6.1^*^
Formaldehyde (μg/cig)	600	636	461	44	52.1	64.5	10.5^*^	^ǂ^7	^ǂ^9.1^*^
Methyl ethyl ketone (μg/cig)	159	120	84.9	81.5	66.4	55.4	35.6	16.6^*^	20.6^*^
Propionaldehyde (μg/cig)	140	108	77.0	59.3	51	43.2	26.4	12.9^*^	14.9^*^
Pyridine (μg/cig)	223	188	136	14.9^*^	20	15.1^*^	3.9	1.2	5.8
Quinoline (μg/cig)	10.7	8.76	6.45	0.4	0.6	0.3	0.2^*^	0.1	0.2^*^
Styrene (μg/cig)	115	85.1	67.3	14.2	12.8	9.5	4.6^*^	1.7	4.4^*^
NAB (ng/cig)	6.68	5.33	4.17	7	5.8^*^	5.4^*^	2.8	1.8^*^	1.8^*^
NAT (ng/cig)	23.3	19.1	12.0	57.2	51.5^*^	44.8^*^	19.7	14.7^*^	14.5^*^
NNK (ng/cig)	156	126	94.9	33.4	23.1^*^	22.3^*^	13.2	7.3^*^	7.1^*^
NNN (ng/cig)	55.9	47.5	31.3	37	29.9^*^	29.2^*^	12.9	7.9^*^	8.9^*^
Catechol (μg/cig)	74.2	56.4	37.9	84.6	72.5	58.5	63.2	30.1^*^	31.6^*^
Hydroquinone (μg/cig)	117	90.7	64.0	90.4	73.8	57.6	52.7	27.7	24.2
m-Cresol (μg/cig)	58.9	42.2	29.7	3.1^*^	3.2^*^	2.8^*^	1.9^*^	0.9	1.7^*^
p-Cresol (μg/cig)				8^*^	8.1^*^	7.4^*^	5.1^*^	1.8	4.6^*^
o-Cresol (μg/cig)	21.1	16.2	11.0	3.4^*^	3.6^*^	3.2^*^	2.1^*^	0.6	2.1^*^
Phenol (μg/cig)	225	169	111	14.4^*^	16^*^	14.2^*^	8.9^*^	2.5	10.2^*^
Resorcinol (μg/cig)	BDL	BDL	BDL	2	1.8	1.5	1.2	0.9	0.6
Nitric oxide (μg/cig)	1987	1653	1460	108.3	86.2	116.6	73.9	46.8	37.2
1,3-Butadiene (μg/cig)	311	247	187	84^*^	80.1^*^	69^*^	52.6	22.1^*^	22.2^*^
Acrylonitrile (μg/cig)	2644	1919	1482	16.9^*^	19^*^	18^*^	8.8	3.3^*^	4.6^*^
Isoprene (μg/cig)	120	89.9	67.7	789.6^*^	779.4^*^	729.7^*^	452.1	227.2^*^	200.8^*^
Benzene (μg/cig)	249	189	141	64.9^*^	60.6^*^	40.6	40.1	18.7^*^	15.7^*^
CO2 (mg/cig)	317	239	168	-	-	-	-	-	-
Toluene (μg/cig)	558	400	296	102.4^*^	84^*^	52.7	54.1	27.7^*^	23.8^*^
NFDPM (HCI-VO) (mg/cig)	-	-	-	17.8	15.8^*^	15.3^*^	7.2	5.1^*^	5.1^*^
Nicotine(HCI-VO) (mg/cig)	-	-	-	1.68^*^	1.67^*^	1.54^*^	0.76	0.49^*^	0.51^*^
CO (HCI-VO) (mg/cig)	-	-	-	14.9	11.3^*^	10.8^*^	6.7	3.6^*^	3.3^*^
Puff no. (HCI-VO)	-	-	-	10	9.1	7.1	10.9	11.2	9
NFDPM (ISO) (mg/cig)	20.8	15.9	10.8	8	7.2^*^	7.1^*^	2.1^*^	1.5^*^	1.5^*^
Nicotine (ISO) (mg/cig)	4.67	3.59	2.43	0.78^*^	^ǂ^0.77^*^	^ǂ^0.71	0.23	0.12^*^	0.14^*^
CO (ISO) (mg/cig)	35.7	26.6	19.8	6.4	5.1^*^	5.3^*^	1.7	0.7^*^	0.7^*^
Puff no. (ISO)	-	-	-	6.8	6.7	5.7	7.6	6.8	6.1

Lack of statistical difference at the 95% confidence level are denoted by ^*^ or ^ǂ^.

Significance testing was also not conducted on puff numbers as these are not discussed in the manuscript.

The impact of altering circumference on the yield of toxicants in mainstream cigarette smoke was examined using 1-mg ISO tar ECs and an equivalent series of 7-mg ISO tar ECs (K711, D711, S711). To meet the specification of 7-mg ISO tar, changes were made to the cigarette paper, filter pressure drop and ventilation levels (Additional file 1: Table S2) in order to balance the effect of tobacco weight on tar yields.

**Figure 5:**
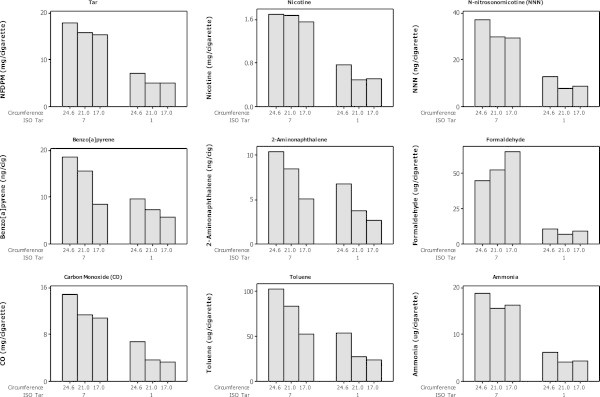
**HCI-VO mainstream yields from 1 and 7-mg ISO tar cigarettes with circumferences between 17.0 and 24.6 mm.**

Mainstream smoke data obtained using HCI-VO smoking parameters for the 1-mg and 7-mg ISO tar cigarettes are presented in Table 3 and Figure 5. ISO tar, nicotine and CO yields were similar among these ECs, although the mainstream ISO tar, nicotine and CO yields of K711 were greater than those of D711 and S711 in each case.

The yields of HCI-VO tar nicotine and CO decreased with reducing circumference, largely in-line with the small changes in ISO yields noted above (Table 3 and Figure 5). The changes in HCI-VO tar across the cigarettes show that the lower tobacco weights of smaller circumference cigarettes produced less mainstream smoke despite the balancing cigarette design factors used to meet target ISO tar specifications.

Mainstream smoke analysis showed that the yields of almost all measured smoke constituents decreased with decreasing circumference, consistent with the changes in tar and CO. With the three 1 mg products ANOVA indicated no significant difference between yields from D111 and S111 for the majority of measured toxicants, although yields from both D111 and S111 were significantly lower than from K111 (p < 0.05) in many cases. In contrast, with the semi-volatile cresols, and phenol, styrene, quinolone, pyridine and HCN significantly lower yields were found from D111 than from K111 and S111. There were no significant differences in formaldehyde yields between these products. For the 7 mg products there were significant increases in formaldehyde yields and significant decreases in CO, aromatic amines, some carbonyls, dihydroxyphenols and benzo[a]pyrene yields with decreasing circumference. A number of toxicants yields were not significantly different between D711 and S111, but lower than from K711. These observations suggested that the composition of smoke might change with cigarette circumference; we therefore examined particulate-phase constituents relative to tar for the 7 mg products. In support of a change in smoke composition, the yields of four aromatic amines and benzo[a]pyrene, NNK and CO decreased more than the tar yield with decreasing cigarette circumference, while formaldehyde yields increased.

Together, these observations show that reducing cigarette circumference not only reduces the quantity of sidestream smoke emissions but also modifies the composition of mainstream smoke (at a fixed tar yield) with increases in formaldehyde and reductions in several particulate- and gas-phase toxicants.

### Influence of HAC loading on vapour-phase constituent yields

The emission of volatile toxicants can be reduced by introducing adsorbents into cigarette filters (Branton et al. 2011a, [b]). We previously reported the extent of reduction in toxicant yields achievable using 60 mg of HAC in a 24.6-mm circumference cigarette, and 20 mg of HAC in a 17-mm circumference cigarette (Branton et al. 2011a). However, it is possible to incorporate higher levels of HAC into cigarette filters (Laugesen and Fowles 2006), which might further increase the removal of volatile toxicants. The level of filter adsorbent can be raised by increasing either the packing density of adsorbent at a fixed filter bed length, or the bed length at a fixed adsorbent packing density. Ultimately, increasing bed-length is a more flexible tool owing to limitations in the amount of adsorbent that can practically fit into a fixed volume of filter without blocking the smoke flow-path and causing unacceptable increases in draw resistance. Furthermore, packing densities differ among cigarettes of different circumference because the internal filter volume reduces with decreasing circumference.

**Figure 6:**
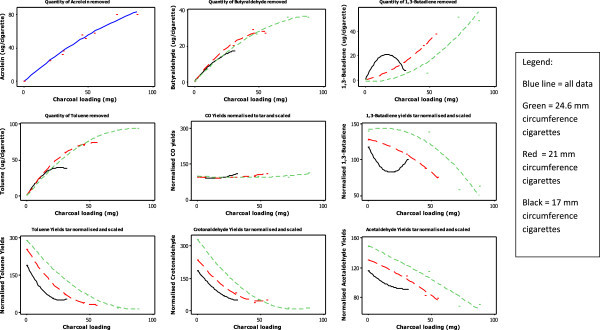
**Influence of HAC loading on yields of volatile toxicants from cigarettes of 17–24.6 mm circumference.**

Mainstream cigarette smoke yields were measured from cigarettes with different HAC loadings (Table 4, and Figure 6). The analytes examined were volatile species that are commonly examined in studies of cigarette filter carbons (Branton et al. 2011a). The HCI-VO tar yields of these cigarettes were matched to within 1 mg (~7%) for each circumference set; however, the data showed that the tar yields of the 24.6- and 21-mm circumference cigarettes decreased systematically as the HAC loading was increased, whereas the tar yield for the two 17-mm circumference cigarettes increased. The differences in tar yields were not large, but their systematic changes should be taken into account in the analysis of toxicant yields. CO and NO were also measured to evaluate the impact of the changed paper specification and levels of split-tipping ventilation in these designs. The data presented above for the split-tipping cigarettes suggested that no significant impact on volatile yields would be expected for the 7-mg ISO tar cigarettes designed for this study; this expectation was confirmed by the measured CO and NO yields, indicating that the experimental design is suitable for the assessment of HAC loading on volatile yields.

**Table 4:** **Effect of HAC loading on mainstream smoke yields of toxicants under HCI-VO smoking conditions**

Variable	Experimental cigarette										
	K715	K712	K721	K731	D711 ^1^	D712	D721	D732	S712	S713	S721
Cigarette ^2^											
HAC loading (mg/cig)	0	48	72	88	0	30	45	55	0	20.4	30.6
Split gap (mm)	0	10	16	20	0	10	16	20	0	10	16
Circumference (mm)	24.6	24.6	24.6	24.6	21.0	21.0	21.0	21.0	17.0	17.0	17.0
Analyte											
Ammonia (μg/cig)	17.6^*^	16.3^*^	16.3^*^	11.5	15.6	14.8^*^	14.7^*^	7.9	17.7	14.1	11.6
Hydrogen cyanide (μg/cig)	163.9	132.2^*^	^ǂ^108.0	^ǂ^118.8^*^	148.7	110.5	94.0	74.0	120.3^*^	97.4	134.9^*^
Acetaldehyde (μg/cig)	611.1	444.1	246.9^*^	260.5^*^	522.9	427.6	314.4^*^	289.4^*^	441.1	361.8^*^	366.2^*^
Acetone (μg/cig)	305.3	152.6	46.1^*^	52.1^*^	251.8	169.1	96.9^*^	98.2^*^	202.6	145. ^*^8	128.3^*^
Acrolein (μg/cig)	91.3	40.6	12.4^*^	11.8^*^	89.0	57.2	34.5^*^	32.3^*^	76.0	52.1	39.9
Butyraldehyde (μg/cig)	42.2	15.6	6.0^*^	5.9^*^	39.3	19.0	10.3^*^	12.0^*^	31.2	17.5	13.9
Crotonaldehyde (μg/cig)	25.8	3.7	0.8^*^	0.7^*^	22.5	7.7	3.2^*^	4.4^*^	20.5	7.9	5.6
Formaldehyde (μg/cig)	45.7	28.2	23.6^*^	23.5^*^	52.1	42.9^*^	^ǂ^36.7^*^	^ǂ^31.9	63.8	51.9	45.1
Methyl ethyl ketone (μg/cig)	73.9	21.4	5.9^*^	6.5^*^	66.4	31.0	15.2^*^	17.0^*^	49.6	26.4	21.3
Propionaldehyde (μg/cig)	55.7	32.5	11.7^*^	13.3^*^	51.0	35.9	22.9^*^	20.9^*^	39.1	30.3^*^	28.5^*^
Pyridine (μg/cig)	9.6	2.1	0.7^*^	0.7^*^	20.0	3.6	1.2^*^	1.3^*^	11.1	3.7	2.1
Quinoline (μg/cig)	0.2	0.3	0.3^*^	0.3^*^	0.6	0.4^*^	0.3	0.4^*^	0.2	0.3	0.2
Styrene (μg/cig)	11.7	1.5	0.4^*^	0.5^*^	12.8	2.0	0.5	0.4	9.0	1.8^*^	1.0^*^
Nitric oxide (μg/cig)	101.9	129.4	115.3	95.9	86.2	83.8^*^	86.3^*^	76.1	^ǂ^74.4^*^	78.1^*^	^ǂ^69.1
1,3-Butadiene (μg/cig)	81.4^*^	76.0^*^	^ǂ^29.9	^ǂ^32.8	80.1	66.2	52.3	42.7	69.8^*^	49.9	62.4^*^
Acrylonitrile (μg/cig)	19.6	11.4	4.2^*^	3.8^*^	19.0	12.2	8.5	6.0	18.9	10.8^*^	10.9^*^
Isoprene (μg/cig)	812.1	572.2	148.6^*^	165.6^*^	779.4	518.3	361.3	251.3	731.0	451.9^*^	500.2^*^
Benzene (μg/cig)	68.5	29.6	6.9^*^	6.6^*^	60.6	27.1	16.4	10.6	42.9	19.2^*^	19.6^*^
Toluene (μg/cig)	98.8	23.5	6.3^*^	4.8^*^	84.0	24.0	13.4	9.4	54.6	17.1^*^	16.4^*^
NFDPM (mg/cig)	18.3^*^	^ǂ^17.2^*^	^ǂ^16.2	^ǂ^16.5	15.8	15.5^*^	15.1^*^	14.4^*^	13.1^*^	13.3^*^	13.8^*^
Nicotine (mg/cig)	1.67^*^	1.60^*^	^ǂ^1.43	^ǂ^1.34	1.67	^ǂ^1.63	^ǂ^1.57	1.36	^ǂ^1.33^*^	1.4^*^	^ǂ^1.25
CO (mg/cig)	14.4^*^	^ǂ^13.6^*^	^ǂ^12.0	14.9^*^	11.3	11.0^*^	11.2^*^	11.9^*^	9.9^*^	9.6^*^	11.6
Puff no.	10.1	9.9	9.7	8.7	9.1	9.2^*^	8.2	10.0^*^	6.8	7.7	6.4
NFDPM (mg/cig)	7.2	8.9^*^	8.4^*^	8.6^*^	7.2	^ǂ^6.8^*^	7.4^*^	^ǂ^6.2	5.8	6.5	7.8
Nicotine (mg/cig)	0.65	0.88^*^	^ǂ^0.78	^ǂ^0.85^*^	0.65	^ǂ^0.71^*^	0.75^*^	^ǂ^0.67	0.56^*^	0.65^*^	0.78
CO (mg/cig)	6.0^*^	6.7^*^	6.8^*^	9.1	6.0	4.7^*^	5.2^*^	5.1^*^	4.4^*^	4.7^*^	7.3
Puff no.	6.9	7.8	6.9	7.0	6.9	6.5	6.2	6.5	5.1	5.5	5.3

^1^Cigarette D711 used a triacetin plasticiser; all other cigarettes used tri-ethyl citrate.

^2^Split-gap sizes changed with cigarette design.

Lack of statistical difference at the 95% confidence level are denoted by ^*^ or ^ǂ^.

Significance testing was not conducted for cigarette code D711 in comparison to D712, D721 or D732 as it‘s construction was slightly different to the other 21 mm cigarettes – it’s data are included for comparative purposes only. Significance testing was also not conducted on puff numbers as these are not discussed in the manuscript.

Overall, the analysis showed that the HCI-VO mainstream smoke yields of many volatile smoke constituents decreased significantly (p < 0.05) as the HAC loading increased (Table 4); toxicants whose yields were reduced included HCN, ammonia, pyridine, acrylonitrile, all measured carbonyls, volatile hydrocarbons and aromatics (e.g. acrolein yields in Figure 6). However, for many of these toxicants, at all three circumferences, the yields of these volatiles at the highest two HAC loadings were not significantly different from each other, indicating a "tailing-off" in the efficiency of HAC at these levels.

As described above, volatile yields were generally reduced for all cigarettes as the HAC loading increased. The compounds that showed the most significant reduction were isoprene, acetaldehyde and acetone, all of which were reduced by more than 250 μg/cigarette at the highest HAC loading. In contrast, much smaller quantities (<25 μg/cigarette) of formaldehyde, pyridine and styrene were removed by the highest HAC loading. These differences arise from a combination of the quantity of each toxicant present in the cigarette smoke entering the filter (in which isoprene, acetaldehyde and acetone are the major constituents by mass concentration) and the efficiency of HAC towards individual smoke constituents.

Some of the observed behaviour in the toxicant yields were clearly influenced by differences in the overall amount of smoke from the different ECs. For example, for the 17-mm circumference ECs, HCN and 1,3-butadiene yields did not change significantly in the 17 mm circumference designs as HAC loading increased. This behaviour paralled changes in the tar yields from the ECs. This influence was removed by normalising the data to the cigarette tar yield in each case. The data were also scaled to a common axis (by setting the mean value of the constituent yields for each circumference series to 100) to allow the removal efficiency of HAC towards different constituents to be examined and compared. Examination of the normalised CO (Figure 6) and NO yields again showed that changes to split-tipping levels and paper permeability had little influence on individual toxicant yields across the three series of cigarettes.

The greatest impact of HAC on tar-normalised and scaled toxicant yields was found with toluene, crotonaldehyde (Figure 6), acrolein, butyraldehyde, methyl ethyl ketone, pyridine, styrene, and benzene, where normalised reductions reached 97% at the highest loadings. For each of these compounds, there was consistent evidence in the data of the diminished efficiency of HAC at the highest loading in each EC. Less extensive percentage reductions were found for other volatile toxicants (e.g., 1,3-butadiene and acetaldehyde in Figure 6), but even for these compounds there was evidence of the diminishing impact of increases in HAC loading. It is noticeable that, for many of the measured constituents across all three circumferences of EC, there was little difference in toxicant removal efficiency between the highest HAC loading in the filters (45–55 mg for the 21-mm cigarettes, and 72–88 mg for the 24.6-mg cigarettes); these observations point to a practical limit in the quantity of HAC that is effective at reducing yields of some toxicants in a cigarette filter.

The study design incorporating cigarette circumferences ranging from 24.6 to 17 mm enabled us to examine the influence of circumference on HAC efficiency towards toxicants. Mixed behaviour was observed for the species measured. For some constituents (acetaldehyde, acetone, acrolein, butyraldehyde, crotonaldehyde, benzene and toluene), the degree of removal by HAC was effectively independent of circumference; that is, common behaviour was exhibited across all three cigarettes (e.g., butyraldehyde and toluene in Figure 6). However, some constituents (formaldehyde, pyridine, styrene, 1,3-butadiene, acrylonitrile and isoprene) showed different behaviour across the three circumferences (e.g. 1,3-butadiene in Figure 6).

Overall, the lowest volatile toxicant yields (both actual and tar-normalised) were found for the highest HAC loading in the 24.6-mm circumference ECs. There were a few cases where either lower or equivalent toxicant yields (HCN, ammonia, pyridine, toluene and styrene) were found for the highest HAC loading in the 21-mm circumference EC, and generally the performance of the 21-mm EC was equivalent to the performance of 60 mg HAC in the larger (24.6 mm) circumference design. In contrast, the volatile toxicant yields from 17-mm circumference cigarettes were substantially higher than those from the 24.6-mm circumference cigarette. This is a consequence of the lower HAC content of these ECs and the shorter residence time of smoke in the filter.

These results show that, in developing a reduced-toxicant cigarette design, a balance must be struck between the lower mainstream benzo[a]pyrene, aromatic amine and CO yields (as well as reduced sidestream yields) from reduced circumference cigarettes, and the increased volatile yields resulting from lower adsorbent incorporation. The mid-point of the circumferences examined in this study, 21 mm, appears to offset these contradictory characteristics in toxicant yield.

### Design and performance characteristics of an RTP cigarette

#### Design

Together with previous studies, the above data demonstrate that several technologies can potentially reduce toxicant emissions from cigarettes in comparison to those from conventional cigarettes. These technologies need to be effectively combined in a cigarette design to prevent unintended increases in toxicant emissions. Once an RTP cigarette is prepared, its performance and relevance as a potentially MRTP need to be evaluated by a series of studies including clinical trials examining biomarkers for toxicant exposure and biological effect. The RTP developed in this study was intended for use in a clinical trial (ISRTCN81286286) in Hamburg, Germany, the protocol of which is described elsewhere (Shepperd et al. 2013b), among German smokers of 6–8 mg ISO yield cigarettes.

Three ECs were manufactured for this study: two versions of the comparator cigarette (differing only in tipping and coding and labelled CC7), and the RTP test product (RTP2). The target ISO tar and nicotine yields for all products were 7.0 and 0.7 mg/cig, respectively. The specification of the comparator cigarettes was based on a popular 7-mg ISO yield British American Tobacco product on sale in Germany at the time of the study, being similar in terms of format (‘King Size’), tobacco blend style (American blend) and filter type (plain cellulose acetate). The comparator cigarette was 83 mm long and 24.6 mm in circumference with a 56-mm tobacco rod and 27-mm single-stage filter. The filter ventilation level was 33% created by OML. The two versions of the comparator product were identical except version 1 used cork tipping paper, whereas version 2 had white tipping paper, a purely visual difference. Because this was a switching study, the two versions enabled maintenance of some level of blinding for the control group.

**Figure 7:**
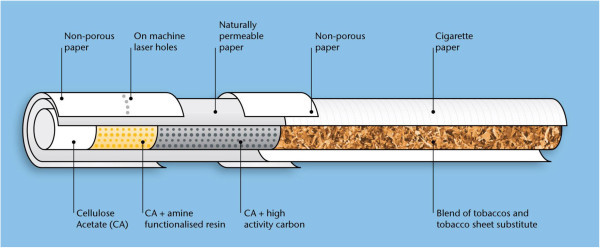
**Schematic showing construction of the RTP2 cigarette.**

The RTP cigarette was developed from available toxicant reduction technologies; the development process involved a series of iterations optimising the chemical and sensory performance of the prototype. Apart from the ISO yields and overall cigarette length, the final test product was substantially different from the comparator (Additional file 1: Table S4). RTP2 had a circumference of 21 mm, and comprised a 46-mm long tobacco rod and a 37-mm filter. The tobacco rod contained a blend of 50% washed, extracted and enzyme-treated tobacco (Liu et al. 2011a), 15% TSS (McAdam et al. 2011) and 35% other tobaccos (Additional file 1: Table S4). The blend content of toxicant precursors (Additional file 1: Table S6) showed lower levels of blend TSNAs, benzo[a]pyrene and most metals. The filter comprised three cellulose acetate segments; the segment nearest the tobacco rod contained 50 mg of HAC (Branton et al. 2011a), the middle segment contained 20 mg of amine-functionalised resin (Branton et al. 2011b), and the mouth-end segment was plain cellulose acetate. Filter ventilation (35%) was achieved by using a split-tipping approach (10-mm wide zone of high-permeability paper), combined with traditional OML ventilation holes. The design of RTP2 is shown in Figure 7.

#### Performance: mainstream and sidestream smoke chemistry

**Figure 8:**
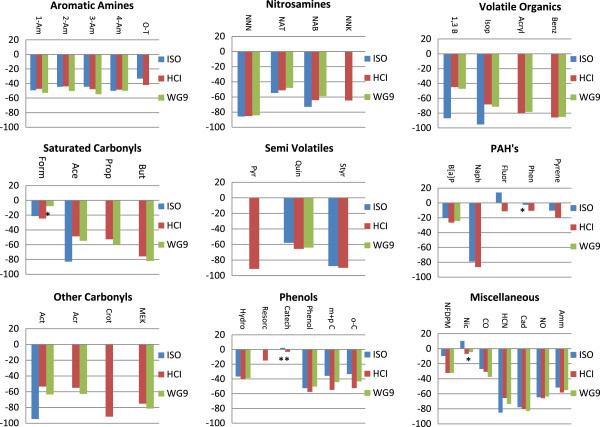
**Comparison of mainstream smoke yields (% difference) from RTP2 in comparison to yields from CC7 under three smoking regimes (ISO, HCI and WG9).**

**Figure 9:**
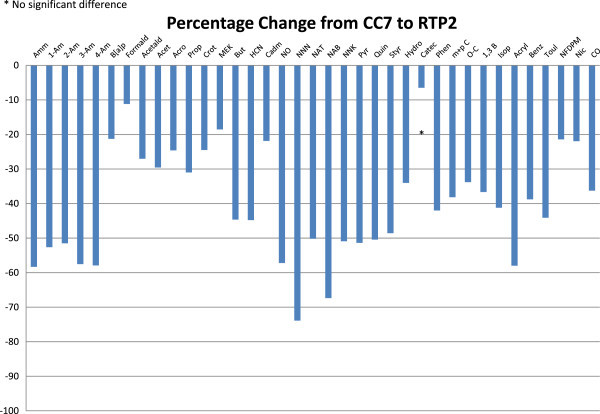
**ISO sidestream smoke yields from RTP2 in comparison to those from CC7 (% basis).**

To quantify their toxicant emissions, the comparator and test cigarettes were smoked under three machine smoking regimes (ISO, WG9, and HCI); the analytes were those examined in a previous publication on RTPs (McAdam et al. 2012) and focused on 44 constituents of regulatory interest (Liu et al. 2011b) plus some constituents corresponding to available biomarkers of exposure (Table 5 and Figures 8 and 9).

**Table 5:** **Comparison of mainstream smoke toxicants between the RTP prototype and a comparative commercial product**

Smoke constituent	CC7			RTP2		
	ISO smoking regime	HCI smoking regime	WG9 smoking regime	ISO smoking regime	HCI smoking regime	WG9 smoking regime
Ammonia (μg/cig)	8.5 ± 0.4	27.8 ± 1.9	29.6 ± 2.3	4.1 ± 0.4	11.6 ± 1.3	13.3 ± 0.8
1-aminonaphthalene (ng/cig)	12.8 ± 0.8	24.4 ± 0.8	23.9 ± 1.4	6.5 ± 0.2	12.9 ± 1.0	11.3 ± 0.8
2-aminonaphthalene (ng/cig)	8.1 ± 0.9	15.5 ± 0.7	16.1 ± 0.9	4.5 ± 0.2	8.7 ± 0.7	8.0 ± 0.6
3-aminobiphenyl (ng/cig)	1.8 ± 0.2	4.2 ± 0.3	4.2 ± 0.2	1.0 ± 0.1	2.2 ± 0.2	1.9 ± 0.1
4-aminobiphenyl (ng/cig)	1.4 ± 0.1	3.1 ± 0.2	3.2 ± 0.3	0.7 ± 0.0	1.6 ± 0.2	1.6 ± 0.1
o-Toluidine (ng/cig)	49.5 ± 1.4	101.3 ± 2.3	NA	33.1 ± 0.4	58.8 ± 4.3	NA
Benzo[a]pyrene (ng/cig)	7.4 ± 0.7	18.5 ± 1.8	17.3 ± 1.8	5.9 ± 0.8	13.6 ± 0.9	13.1 ± 0.7
Formaldehyde (μg/cig)	21.5 ± 2.3	64.4 ± 7.9	54.1 ± 10.8	16.9 ± 1.7	48.6 ± 4.3	49.9 ± 5.3
Acetaldehyde (μg/cig)	393.2 ± 30.1	1121.7 ± 39.5	959.1 ± 79.6	67.1 ± 12.5	576.1 ± 36.4	434.3 ± 33.4
Acetone (μg/cig)	198.2 ± 13.1	550.6 ± 16.2	479.4 ± 35.8	10.6 ± 2.2	256.4 ± 28.2	175.4 ± 7.4
Acrolein (μg/cig)	43.6 ± 5.6	137.0 ± 5.3	120.6 ± 13.1	NQ	61.6 ± 5.5	44.8 ± 2.9
Propionaldehyde (μg/cig)	36.4 ± 3.2	102.8 ± 2.8	89.2 ± 7.8	NQ	48.8 ± 4.2	35.6 ± 1.8
Crotonaldehyde (μg/cig)	9.5 ± 1.3	46.9 ± 1.9	37.4 ± 5.2	BDL	3.9 ± 1.4	NQ
Methyl ethyl ketone (μg/cig)	48.5 ± 3.8	141.0 ± 3.8	121.8 ± 10.7	NQ	35.0 ± 7.0	22.8 ± 0.8
Butyraldehyde (μg/cig)	26.3 ± 2.3	69.7 ± 2.8	65.1 ± 6.3	BDL	16.9 ± 2.0	11.7 ± 0.9
HCN (μg/cig)	84.8 ± 4.0	316.3 ± 12.3	304.6 ± 19.6	12.7 ± 1.1	109.0 ± 2.7	80.6 ± 9.8
Mercury (ng/cig)	NQ	3.8 ± 0.4	NQ	BDL	NQ	NQ
Cadmium (ng/cig)	14.5 ± 0.8	48.7 ± 1.2	47.3 ± 1.8	3.2 ± 0.2	9.7 ± 0.5	8.1 ± 0.8
Lead (ng/cig)	NQ	NQ	NQ	BDL	NQ	BDL
Chromium (ng/cig)	BDL	BDL	BDL	BDL	BDL	BDL
Nickel (ng/cig)	BDL	BDL	BDL	BDL	BDL	BDL
Arsenic (ng/cig)	NQ	NQ	NQ	NQ	NQ	NQ
Selenium (ng/cig)	BDL	BDL	BDL	BDL	BDL	BDL
NO (μg/cig)	88.5 ± 4.7	256.5 ± 32.6	234.1 ± 14.4	31.3 ± 2.9	87.4 ± 10.7	85.0 ± 9.0
NNN (ng/cig)	69.5 ± 6.8	171.4 ± 8.2	143.9 ± 9.2	9.9 ± 1.0	25.4 ± 2.0	22.8 ± 2.8
NAT (ng/cig)	47.4 ± 2.1	114.5 ± 2.7	95.8 ± 3.2	21.5 ± 1.6	55.9 ± 4.2	49.8 ± 5.7
NAB (ng/cig)	8.9 ± 1.1	18.2 ± 1.4	15.0 ± 1.0	2.4 ± 0.3	6.5 ± 0.3	6.2 ± 0.6
NNK (ng/cig)	32.6 ± 3.7	79.9 ± 2.9	70.9 ± 4.5	NQ	28.3 ± 2.8	NQ
Pyridine (μg/cig)	8.3 ± 0.3	36.7 ± 2.5	33.6 ± 0.9	NQ	3.2 ± 0.9	NQ
Quinoline (μg/cig)	0.19 ± 0.01	0.35 ± 0.02	0.39 ± 0.02	0.08 ± 0.01	0.12 ± 0.01	0.14 ± 0.01
Styrene (μg/cig)	5.7 ± 0.3	23.3 ± 1.4	20.5 ± 0.6	0.7 ± 0.2	2.3 ± 0.6	NQ
Hydroquinone (μg/cig)	43.8 ± 1.9	113.1 ± 2.9	108.9 ± 7.3	27.7 ± 1.6	67.7 ± 5.9	66.7 ± 4.5
Resorcinol (μg/cig)	NQ	2.0 ± 0.2	2.5 ± 0.2	BDL	1.7 ± 0.2	NQ
Catechol (μg/cig)	48.4 ± 2.4	99.5 ± 7.6	102.8 ± 6.5	49.4 ± 3.5	96.4 ± 8.8	103.6 ± 6.9
Phenol (μg/cig)	9.5 ± 0.6	14.6 ± 1.7	17.1 ± 3.1	4.5 ± 0.9	6.2 ± 0.7	8.5 ± 0.7
m + p cresols (μg/cig)	6.7 ± 0.4	11.5 ± 1.2	13.1 ± 1.5	4.3 ± 0.5	5.2 ± 0.4	7.3 ± 0.6
o-cresol (μg/cig)	2.4 ± 0.2	3.8 ± 0.4	4.6 ± 0.5	1.6 ± 0.2	1.8 ± 0.2	2.6 ± 0.2
1,3-butadiene (μg/cig)	33.0 ± 1.1	95.3 ± 3.5	89.9 ± 5.0	4.3 ± 0.6	52.5 ± 2.8	47.6 ± 5.0
Isoprene (μg/cig)	283.8 ± 16.7	817.0 ± 21.8	771.1 ± 36.8	13.1 ± 2.5	259.5 ± 18.3	221.9 ± 29.7
Acrylonitrile (μg/cig)	5.6 ± 0.7	23.3 ± 3.5	19.9 ± 3.5	BDL	4.7 ± 0.7	4.3 ± 0.9
Benzene (μg/cig)	26.6 ± 2.8	68.5 ± 3.9	63.6 ± 6.2	BDL	9.7 ± 1.1	9.5 ± 1.3
Toluene (μg/cig)	44.1 ± 5.2	127.6 ± 9.1	120.5 ± 12.7	NQ	NQ	NQ
Naphthalene (ng/cig)	257.6 ± 19.1	1048.5 ± 38.1	NA	54.1 ± 3.6	142.0 ± 9.1	NA
Fluorene (ng/cig)	141.4 ± 8.6	269.2 ± 8.6	NA	161.5 ± 4.6	238.8 ± 15.9	NA
Phenanthrene (ng/cig)	99.7 ± 8.2	182.4 ± 6.7	NA	97.3 ± 2.7	163.2 ± 10.1	NA
Pyrene (ng/cig)	41.5 ± 2.0	88.3 ± 2.7	NA	37.2 ± 1.2	70.9 ± 3.6	NA
NFDPM (mg/cig)	7.1 ± 0.4	26.3 ± 1.7	23.9 ± 2.4	6.4 ± 0.3	17.8 ± 1.2	16.2 ± 0.5
Nicotine (mg/cig)	0.58 ± 0.04	1.59 ± 0.04	1.52 ± 0.14	0.64 ± 0.03	1.48 ± 0.06	1.45 ± 0.04
CO (mg/cig)	7.4 ± 0.4	22.7 ± 1.1	22.4 ± 0.8	5.4 ± 0.3	15.7 ± 0.8	14.0 ± 0.3

*Abbreviations:* *BDL* below detection limit, *NA* not available, *NQ* not quantified.

Values are given as mean ± SD; 5 replicates.

Under all three smoking regimes, the mainstream smoke yields of all toxicants examined other than nicotine, fluorene, phenanthrene and catechol, were lower from the RTP cigarette than from the comparator product (Figures 8 and 9). Under the ISO smoking regime, the yields of some toxicants were below the analysis reporting limit, but other than this there was a good deal of consistency in the directional changes across the three smoking regimes. In comparison to the comparator product, the RTP cigarette showed small differences (<25%) in PAHs (including benzo[a]pyrene), formaldehyde and resorcinol yields. More substantial reductions in yields (<50%) were found for CO, aromatic amines, 1,3-butadiene, and most phenols. The yields of most TSNAs, carbonyls, NO, and ammonia were more than 50% lower from the RTP than from the comparator product, whereas the yields of NNN, and volatile species such as HCN, pyridine, acrylonitrile, 1,3-butadiene, benzene, styrene, naphthalene, methyl ethyl ketone and cadmium were more than 80% less as compared with the comparator cigarette. The RTP’s mainstream smoke toxicant profile showed more balanced reductions across the range of measured toxicants than found previously for earlier prototype designs (McAdam et al. 2011), achieving one of the main design goals of this study. In particular, the current design showed superior reductions of isoprene, phenols and benzo[a]pyrene as compared with prototype BT1, and superior reductions of aromatic amines as compared with RTP2.

Sidestream yields were also measured under ISO smoking parameters (Table 6 and Figure 9). Apart from some of the metals, whose yields were below method reporting limits, and catechol, for which significance was not reached, sidestream yields of all toxicants were lower from RTP2 than from the commercial comparator cigarette. The reductions ranged from approximately 10% for formaldehyde to over 70% for NNN; the predominance of reductions covered the range 20%–60%.

**Table 6:** **Comparison of sidestream smoke chemistry between the RTP prototype and a comparative commercial product**

Smoke constituent	CC7	RTP2
	ISO smoking regime	ISO smoking regime
Ammonia (μg/cig)		
1-aminonaphthalene (ng/cig)	194.3 ± 7.9	92.0 ± 6.0
2-aminonaphthalene (ng/cig)	158.2 ± 4.2	76.7 ± 4.4
3-aminobiphenyl (ng/cig)	35.1 ± 1.3	14.9 ± 1.1
4-aminobiphenyl (ng/cig)	24.0 ± 1.5	10.1 ± 0.7
Benzo[a]pyrene (ng/cig)	118.9 ± 11.7	93.6 ± 5.4
Formaldehyde (μg/cig)	590 ± 92	524 ± 31
Acetaldehyde (μg/cig)	1535 ± 114	1120 ± 130
Acetone (μg/cig)	832 ± 60	586 ± 53
Acrolein (μg/cig)	339.3 ± 27.1	255.8 ± 32.6
Propionaldehyde (μg/cig)	158.5 ± 10.2	109.4 ± 8.8
Crotonaldehyde (μg/cig)	75.5 ± 5.7	57.0 ± 3.9
Methyl ethyl ketone (μg/cig)	200.3 ± 13.3	163.2 ± 13.7
Butyraldehyde (μg/cig)	97.0 ± 4.8	53.7 ± 4.2
HCN (μg/cig)	97.3 ± 10.1	53.7 ± 7.8
Mercury (ng/cig)	NQ	NQ
Cadmium (ng/cig)	207.6 ± 7.5	162.2 ± 10.0
Lead (ng/cig)	BDL	BDL
Chromium (ng/cig)	NQ	NQ
Nickel (ng/cig)	NQ	NQ
Arsenic (ng/cig)	BDL	BDL
Selenium (ng/cig)	BDL	BDL
NO (μg/cig)	2064 ± 62	883 ± 101
NNN (ng/cig)	89.9 ± 11.1	23.5 ± 1.3
NAT (ng/cig)	30.3 ± 3.7	15.1 ± 0.8
NAB (ng/cig)	9.8 ± 0.6	3.2 ± 0.3
NNK (ng/cig)	149.3 ± 8.9	73.3 ± 3.9
Pyridine (μg/cig)	269.9 ± 10.7	131.2 ± 7.7
Quinoline (μg/cig)	11.7 ± 0.4	5.8 ± 0.4
Styrene (μg/cig)	94.7 ± 6.4	48.7 ± 5.2
Hydroquinone (μg/cig)	93.5 ± 11.6	61.7 ± 2.6
Resorcinol (μg/cig)	BDL	BDL
Catechol (μg/cig)	71.1 ± 7.4	66.5 ± 4.4
Phenol (μg/cig)	225.6 ± 10.5	130.8 ± 6.2
m + p cresol (μg/cig)	73.6 ± 3.2	45.5 ± 3.0
o-cresol (μg/cig)	35.5 ± 1.5	23.5 ± 1.8
1,3-butadiene (μg/cig)	412 ± 40	261 ± 21
Isoprene (μg/cig)	3041 ± 248	1788 ± 116
Acrylonitrile (μg/cig)	119.7 ± 15.9	50.3 ± 8.0
Benzene (μg/cig)	281 ± 26	172 ± 19
Toluene (μg/cig)	558 ± 33	312 ± 28
NFDPM (mg/cig)	21.0 ± 1.4	16.5 ± 0.6
Nicotine (mg/cig)	4.01 ± 0.14	3.13 ± 0.14
CO (mg/cig)	40.0 ± 3.4	25.5 ± 2.9
CO_2_ (mg/cig)	305 ± 26	196 ± 11

*Abbreviations:* *BDL* below detection limit, *NA* not available, *NQ* not quantified.

Values are given as mean ± SD, 5 replicates.

Taken together, these data show that the development of this RTP cigarette achieved the aim of producing a cigarette design that offers lower smoke toxicant yields as compared with a reference commercial cigarette design.

## Discussion

We have reported a new approach to toxicant reduction from cigarettes, termed split-tipping, together with fresh insights concerning the potential for using cigarette circumference and longer filters containing higher than conventional levels of HAC adsorbent for decreasing a wide range of toxicant emissions from cigarettes.

Our data showed that the split-tipping filter ventilation approach is a functional mechanism for minimising the reduction in effective ventilation levels that occurs at puffing flow rates higher than those of the ISO smoking regime. Split-tipping was found to be most effective at high ventilation levels (i.e. low ISO tar cigarettes) and high flow rates; it had little impact on product performance for the higher ISO tar cigarettes in this study. Amongst humans, split-tipping led to significant reductions in MLE for smokers of 1-mg tar products but not for smokers of higher tar cigarettes. Regarding the individual toxicant yields from split-tipping cigarettes under machine-smoking conditions, reductions in particulate-phase toxicant yields were found to follow tar; however, more substantial reductions were observed for some volatiles, probably due to diffusion through the paper wrapper as the smoke travels down the tobacco rod during a puff, or through the broad permeable split-gap paper in the filter. These observations were made on 24.6-mm king-sized cigarettes; for smaller circumference cigarettes, the effective flow rate though the product is faster and it is possible that greater effectiveness may be observed for cigarettes with a circumference smaller than 24.6 mm.

Our evaluation of the influence of cigarette circumference on toxicant emissions showed significant impact. Sidestream measurements under controlled conditions demonstrated progressive reductions in all measured smoke emissions, covering particulate, gaseous, nitrogenous and carbon based toxicants, other than formaldehyde, with the decrease in tobacco weight burnt. This reflects a general reduction in quantities of precursors for the smoke toxicants as circumference and tobacco weight reduce. The formaldehyde results are consistent with three somewhat competitive influences on formaldehyde yields: i) the reduction in quantity of precursors such as sugars and cellulosic materials acting to reduce the formaldehyde emissions, ii) the reduction in sidestream ammonia yields inhibiting the energetically favourable reaction of ammonia with formaldehyde to form hexamethylenetetramine, thereby potentially boosting formaldehyde emissions, and iii) greater oxidative formation of formaldehyde as the circumference is reduced and surface area to internal volume increases. Comparison with data from the uncontrolled-design market-survey study of Canadian cigarettes from 2004 showed that other factors (e.g. blend content of TSNAs and other species) also affect the sidestream yields of some toxicants.

The data also indicated that the balance of mainstream smoke composition alters as the circumference changes. Moving to a smaller circumference cigarette significantly reduces the relative smoke content of pyrolytically generated species such as benzo[a]pyrene, aromatic amines, and CO but increases the content of species arising from more oxidative burning such as formaldehyde. Similar data were recently obtained for Canadian cigarettes (ISO 3308 2000), where reductions in aromatic amines, benzo[a]pyrene, carbonyls, volatiles, CO, and increases in formaldehyde and ammonia yields were observed for superslim cigarettes in comparison to the Canadian Benchmark dataset. The Canadian study was necessarily observational rather than controlled; as a result, many differences in blend composition and cigarette design in addition to circumference would be encompassed by the observations (ISO 3308 2000). For example, if the change from normal circumference to small circumference cigarettes were accompanied by more US-blended character in the slimmer cigarettes, then changes of the kind seen in the Canadian study could be expected. Overall, the general agreement between the findings from market samples (ISO 3308 2000) and those from the present controlled comparison is encouraging.

The findings may be explained through two possible related mechanisms. First, the reduced levels of volatile toxicants are likely to be a consequence of the smaller ratio of internal volume to paper surface area in reduced circumference cigarettes, facilitating greater diffusional losses through the cigarette paper as the smoke travels down the tobacco rod during a puff. The smaller ratio is also likely to change the balance of oxygen to tobacco within the cigarette; that is, there may be greater oxygen ingress to the burning coal in a reduced circumference cigarette. These changes might alter the effective combustion stoichiometry in small circumference cigarettes, leading to greater oxidative burning and reduced formation of pyrolytic products.

The experiments examining the impact of carbon loading on toxicant yields confirmed that substantial decreases in the emissions of mainstream volatile constituents can be obtained at high HAC loading. For the small aromatic toxicants, some of the carbonyls and pyridine, the percentage removal by the highest loadings of HAC reached 97%, demonstrating the effectiveness of HAC for these toxicants. In contrast, somewhat lower removal of isoprene (82%), acetaldehyde (60%) and acetone (85%) was observed, although the amounts removed were very high (250–650 μg/cigarette). The total amount removed of these three toxicants alone was approximately 1.5% of the HAC mass, demonstrating the significant adsorption potential of the HAC material.

For many volatile toxicants, there appeared to be a tailing-off in adsorption performance at the higher HAC loading of each cigarette. This is likely to be a reflection of the physical chemistry of individual toxicants (e.g. volatility, or particle-vapour partitioning within smoke), combined with the effectiveness of HAC in removing physically available toxicants within the smoke stream. For example, we have previously noted the challenge of removing very volatile species (e.g. 1,3-butadiene and ammonia) from smoke with HAC owing to the high vapour pressures of these species at the flow rates and temperatures in cigarette filters (Branton et al. 2011a). Formaldehyde and HCN yields were reduced by less than 50% at the highest levels of HAC in the present study. Previous work has demonstrated that both species are partitioned between vapour and particulate phases in mainstream smoke (Baker 1999, 2006), thereby limiting their availability to adsorbents; this constrains the amounts that can be removed from mainstream smoke (Branton et al. 2011b).

Some sensitivity of toxicant adsorption by HAC to cigarette circumference was observed. The effectiveness of an adsorbent such as HAC is likely to be weaker in smaller circumference cigarettes because, at a fixed puff volume, the flow rate of air through the cigarette is increased at smaller circumferences owing to the reduced volume. Hence, smoke passing through a filtration bed will have a shorter residence time in the filtration media and removal efficiency of volatiles by physico-adsorption will be reduced. The effect of residence time on chemisorption of mainstream smoke toxicants by the chemisorbent CR20 has been discussed previously, wherein kinetic analysis demonstrated that a fixed CR20 bed has diminished toxicant stripping properties under enhanced flow rate conditions (Branton et al. 2011b). Counteracting the reduced adsorbent efficiency of smaller circumference cigarettes is the apparent increased diffusional loss of volatile species through the cigarette paper observed in the current work. For each toxicant, there will be an individual balance between its diffusional characteristics through cigarette paper and its adsorptivity on HAC, and these competing factors explain the various behaviours at different circumferences seen in this study.

On the basis of the present data, it is clear that reduced levels of sidestream toxicants and mainstream pyrolytic toxicants can be readily achieved with lower cigarette circumference, albeit at the expense of increased formaldehyde yields. Increasing HAC loading further reduced the yields of volatile toxicants, although the loading capacity was limited at the smallest circumference cigarettes. Combining these features led us to an optimum cigarette design (given the limitations of available manufacturable cigarette formats) for a reduced toxicant prototype with a 21-mm circumference and a three-segment filter containing 55 mg of HAC and 20 mg of CR20 (to address the substantial increases in formaldehyde emissions (Branton et al. 2011b)). The split-tipping technology was incorporated to counteract lost diffusional opportunities for volatile constituents due to the shorter cigarette rod and paper length, as well as adding the potential for small reductions in MLE associated with the higher effective flow rate in the 21-mm cigarette. The tobacco blend for this prototype incorporated TSS, BTT and low-toxicant tobaccos.

Mainstream and sidestream smoke yield data showed that, in comparison to the commercial comparator cigarette, the RTP2 cigarette achieved the aim of producing a cigarette design that offers lower smoke yields across a range of toxicants with no significant increase in any of the toxicants measured.

## Conclusions

The work reported in this paper extends the range of available technological approaches for reducing emissions of cigarette smoke toxicants. In addition to a newly reported, "split-tipping" approach to enhance mainstream smoke air dilution, we have identified features of cigarette design, such as circumference and adsorbent loading, that can be optimised to reduce a broad range of toxicant emissions. Combining these features with a tobacco blend comprising low toxicant tobacco, a glycerol-loaded TSS, tobacco treated to remove precursors of nitrogenous and phenolic toxicants, and two different filter adsorbents produced a prototype EC with significantly lower mainstream and sidestream toxicant yields than those from a commercial comparator cigarette. These observations warrant further evaluation in clinical studies where the relevance of these observations may be tested using biomarkers of exposure and physiological effect. These studies will be reported separately.

## Electronic supplementary material

Additional file 1: **Table S1.** Design and specifications of experimental cigarettes used in the split–tipping study. **Table S2.** Design and specifications of experimental cigarettes used in the circumference study. **Table S3.** Design and specifications of experimental cigarettes used in the HAC adsorbent loading study. **Table S4.** Design and specifications of the RTP prototype and commercial comparator cigarettes. **Table S5.** Analytical methods and reporting limits for analysis of blend analytes, and mainstream and sidestream smoke toxicants. **Table S6.** Blend chemistry of experimental cigarettes. (PDF 189 KB)
