# Supplementary material for: Approaches for the design of reduced toxicant emission cigarettes
Source: Springerplus. 2014 Jul 22;3:374. doi: 10.1186/2193-1801-3-374 (PMC4125608; doi:10.1186/2193-1801-3-374)
Supplement: Supplementary file 1 — Additional file 1: Table S1. Design and specifications of experimental cigarettes used in the split–tipping study. Table S2. Design and specifications of experimental cigarettes used in the circumference study. Table S3. Design and specifications of experimental cigarettes used in the HAC adsorbent loading study. Table S4. Design and specifications of the RTP prototype and commercial comparator cigarettes. Table S5. Analytical methods and reporting limits for analysis of blend analytes, and mainstream and sidestream smoke toxicants. Table S6. Blend chemistry of experimental cigarettes. (PDF 189 KB) [file 40064_2014_1094_MOESM1_ESM.pdf]

Supplementary Tables

Table S1. Design and specifications of experimental cigarettes used in the split-tipping study

Test type	ISO tar specification (mg/cig)	Paper permeability (CU)	Tobacco rod characteristics	Filter characteristics	Filter ventilation	Target ventilation (%)	Split-gap paper porosity (CU)
Control	1	62	83 mm length, 24.6 mm circumf. USB blend A	27 mm CA single segment filter, 7% triacetin loading	OML	82	
Split		64			ST[1]		1000
Control	4	57			OML	62	
Split		58			ST[1]		500
Control	7	60	83 mm length, 24.6 mm circumf. USB blend B	27 mm CA single segment filter, 7% triacetin loading	OML	45	
Split		54			ST[1]		240
Control	10	56			OML	28	
Split		65			ST[1]		110

Abbreviations: CA, cellulose acetate; CU, CORESTA units ; OML, on-machine laser; ST, split-tipping ; USB, US style tobacco blend.

[1] Split-tipping with porous paper split-gap (adjusted to target ventilation with OML).

Table S2. Design and specifications of experimental cigarettes used in the circumference study

Cigarette code	K111	D111	S111	K711	D711	S711
Tobacco						
Blend style	MFCV	MFCV	MFCV	MFCV	MFCV	MFCV
Blend technology	TSS	TSS	TSS	TSS	TSS	TSS
Technology inclusion (%)	20	20	20	20	20	20
Top flavour addition (w/w%)	-	-	-	-	-	-
Rod length (mm)	56	56	56	56	56	56
Density (mg/cc)	211	215	242	235	240	232
Blend weight (mg)	540	397	297	584	445	301
Moisture (%)	12.7	12.8	12.5	13.5	13.1	13.2
Filter						
Format	Mono	Mono	Mono	Mono	Mono	Mono
Filter length (mm)	27	27	27	27	27	27
Mouth end segment	27mm CA	27mm CA	27mm CA	27mm CA	27mm CA	27mm CA
Middle segment	-	-	-	-	-	-
Tobacco end segment	-	-	-	-	-	-
Plasticiser type	TA	TA	TA	TA	TA	TA
Filter PD (mmWG)	110	142	182	84	104	145
Filter ventilation (%)	79	84	82	42	41	21
Split tipping	No	No	No	No	No	No
Split tip vent zone/gap (mm)	-	-	-	-	-	-
OML	Yes	Yes	Yes	Yes	Yes	Yes

Cigarette						
Total length (mm)	83	83	83	83	83	*83*
Circumference (mm)	24.6	21.0	17.0	24.6	21.0	17.0
Total weight (mg)	791	617	454	835	654	454
Paper permeability (CU)	50	50	25	50	50	25
Paper citrate level (%)	1	1	1	1	1	1
ISO NFDPM target (mg)	1	1	1	7	7	7

Abbreviations: CA, cellulose acetate; CU, CORESTA units; NFDPM, nicotine-free dry particulate matter.

Table S3. Design and specifications of experimental cigarettes used in the HAC adsorbent loading study

Cigarette code	K712	D712	S713	K715	K721	K731	D721	D732	S712	S721
Tobacco										
Blend style	MFCV	MFCV	MFCV	MFCV	MFCV	MFCV	MFCV	MFCV	MFCV	MFCV
Blend technology	TSS	TSS	TSS	TSS	TSS	TSS	TSS	TSS	TSS	TSS
Technology inclusion (%)	20	20	20	20	20	20	20	20	20	20
Top Flavour addition (w/w%)	-	-	-	-	-	-	-	-	-	-
Rod length (mm)	56	56	56	56	56	56	56	56	56	56
Density (mg/cc)	258	242	252	231	237	232	226	225	250	240
Blend weight (mg)	642	453	304	587	532	483	384	350	304	252
Moisture (%)	13.0	12.7	13.1	13.0	12.7	12.2	13.0	13.2	12.4	12.4
Filter										
Format	Dual	Dual	Dual	Mono	Dual	Dual	Dual	Dual	Mono	Dual
Filter length (mm)	27	27	27	27	33	37	33	37	27	33
Mouth end segment	15mm CA	15mm CA	15mm CA	27mm CA	15mm CA	15mm CA	15mm CA	15mm CA	27mm CA	15mm CA
Middle segment	-	-	-	-	-	-	-	-	-	-
Tobacco end segment	12mm CA + 48mg HAC	12mm CA + 30mg HAC	12mm CA + 20.4mg HAC	-	18mm CA + 45.0mg HAC	22mm CA + 88.0mg HAC	18mm CA + 45.0mg HAC	22mm CA + 55.0mg HAC	-	18mm CA + 30.6mg HAC
Plasticiser type	TEC	TEC	TEC	TEC	TEC	TEC	TEC	TEC	TEC	TEC
Filter PD (mmWG)	80	92	139	106	74	81	95	97	148	146
Filter ventilation (%)	39	39	27	41	42	40	39	49	22	31
Split tipping	Yes	Yes	Yes	No	Yes	Yes	Yes	Yes	No	Yes
Split tip vent zone/gap (mm)	10	10	10	-	16	20	16	20	-	16
OML	Yes	Yes	Yes	Yes	Yes	Yes	Yes	Yes	Yes	Yes

Cigarette										
Total length (mm)	*83*	*83*	*83*	83	83	83	83	83	83	83
Circumference (mm)	24.6	21.0	17.0	24.6	24.6	24.6	21.0	21.0	17.0	17.0
Total weight (mg)	943	702	474	834	875	874	677	665	469	452
Paper permeability (CU)	75	75	50	50	25	5 (+EP50)	25	5 (+EP50)	25	5 (+EP50)
Paper citrate level (%)	1	1	0.6	1	0.6	3	0.6	1	1	3
ISO NFDPM target (mg)	7	7	7	7	7	7	7	7	7	7

Abbreviations: CA, cellulose acetate; CU, CORESTA units; NFDPM, nicotine-free dry particulate matter.

Table S4. Design and specifications of the RTP prototype and commercial comparator cigarettes

Cigarette Code	CC7	RTP2
Tobacco		
Blend style	USB	MFCV
Blend technology	-	BTT/TSS
Technology inclusion (%)	-	50/15
Top flavour addition (w/w%)	-	0.8
Rod length (mm)	56	46
Density (mg/ml)	231	312
Blend weight (mg)	598	490
Moisture (%)	13.3	12.6
Filter		
Format	Mono	Triple
Filter length (mm)	27	37
Mouth end segment	27mm CA	7mm CA
Middle segment	-	10mm CA + 20mg AFR
Tobacco end segment	-	20mm CA + 50mg HAC
Filter PD (mmWG)	87	113
Filter ventilation (%)	33	35
Split tipping	No	Yes
Split tip vent zone/gap (mm)	-	10
OML	Yes	Yes

Cigarette		
Total length (mm)	83	83
Circumference (mm)	24.6	21.0
Total weight (mg)	830	830
Paper permeability (CU)	75	50
Paper citrate level (%)	2	1
End stability (mg/end)	1.7	4.7
ISO NFDPM target (mg)	7	7

Abbreviations: CA, cellulose acetate; CU, CORESTA units; NFDPM, nicotine-free dry particulate matter.

Table S5. Analytical methods and reporting limits for analysis of blend analytes, and mainstream and sidestream smoke toxicants

Analysis approach	Mainstream smoke		Mainstream smoke		Sidestream smoke		Tobacco blend	
Analytical lab	British American Tobacco		Labstat International		Labstat International		British American Tobacco	
Functional group and analyte	BAT method*	Reporting limit[a] (Units)	Health Canada method	Reporting limit[b] (Units)	Health Canada method	Reporting limit[b] (units)	BAT method	Reporting limit[a] (units)
Ammonia	S002N (AMD-1601)	0.80 µg/cig	T-101	2.44 µg/cig	T-201	213 µg/cig		
**Aromatic Amines**	S064U (AMD-1642)		T-102		T-202			
1-Aminonaphthalene		0.45 ng/cig		0.425 ng/cig		2.13 ng/cig		
2-Aminonaphthalene		0.45 ng/cig		0.552 ng/cig		2.76 ng/cig		
3-Aminobiphenyl		0.09 ng/cig		0.069 ng/cig		0.34 ng/cig		
4-Aminobiphenyl		0.09 ng/cig		0.069 ng/cig		0.34 ng/cig		
Benzo[a]pyrene	S015U (AMD-1609)	1.25 ng/cig	T-103	0.704 ng/cig	T-203A	3.35 ng/cig	T115U (AMD-2140)	0.25 ng/g
**Carbonyls**	S017U (AMD-1611)		T-104		T-204			
Formaldehyde		0.85 µg/cig		1.20 µg/cig		2.11 µg/cig		
Acetaldehyde		1.15 µg/cig		3.24 µg/cig		5.68 µg/cig		
Acetone		1.45 µg/cig		2.82 µg/cig		4.94 µg/cig		
Acrolein		0.95 µg/cig		2.38 µg/cig		4.16 µg/cig		
Propionaldehyde		0.95 µg/cig		3.34 µg/cig		5.84 µg/cig		
Crotonaldehyde		1.10 µg/cig		3.29 µg/cig		5.76 µg/cig		
Methyl Ethyl Ketone		1.10 µg/cig		3.66 µg/cig		6.41 µg/cig		
Butyraldehyde		1.15 µg/cig		2.71 µg/cig		4.74 µg/cig		
**Hydrogen Cyanide**	S018U (AMD-1612)	5.60 µg/cig	T-107	1.75 µg/cig	T-205	13.1 µg/cig		
**Metals**	S066N (AMD-1643)		T-109		T-207		AMD-1644	

Cadmium		1.93 ng/cig		1.59 ng/cig		13.4 ng/cig		0.02 µg/g
Lead		12.03 ng/cig		12.8 ng/cig		115 ng/cig		0.02 µg/g
Chromium		1.17 ng/cig		19.8 ng/cig		103 ng/cig		0.04 µg/g
Nickel		1.99 ng/cig		21.6 ng/cig		118 ng/cig		0.08 µg/g
Arsenic		0.97 ng/cig		3.75 ng/cig		37.7 ng/cig		0.02 µg/g
Selenium		4.10 ng/cig		7.37 ng/cig		58.4 ng/cig		0.02 µg/g
Mercury	S066N (AMD-1643)	0.13 ng/cig	T-108	1.43 ng/cig	T-206	6.00 ng/cig	AMD-1644	0.13 µg/g
**Oxides of Nitrogen**			T-110		T-208			
NO	S185N (AMD-1669)	40 µg/cig		2.70 µg/cig		186 µg/cig		
NOx				7.52 µg/cig		179 µg/cig		
**Tobacco Specific Nitrosamines**	S168U (AMD-1664)		T-111		T-209		AMD-1646	
N-Nitrosonornicotine (NNN)		0.18 ng/cig		4.95 ng/cig		6.19 ng/cig		0.0018 µg/g
N-Nitrosoanatabine (NAT)		0.18 ng/cig		6.25 ng/cig		7.81 ng/cig		0.0018 µg/g
N-Nitrosoanabasine (NAB)		0.09 ng/cig		2.11 ng/cig		2.64 ng/cig		0.009 µg/g
4-(N-nitrosomethylamino)-1-(3-pyridyl)-1-butanone (NNK)		0.18 ng/cig		12.4 ng/cig		15.5 ng/cig		0.0018 µg/g
**(Semi-Volatiles)**	S020U (AMD-1614)		T-112		T-210			
Pyridine		0.80 µg/cig		0.791 µg/cig		9.89 µg/cig		
Quinoline		0.08 µg/cig		0.024 µg/cig		0.30 µg/cig		
**Phenols**	S019U (AMD-1613)		T-114		T-211			
Hydroquinone		3.20 µg/cig		3.09 µg/cig		14.1 µg/cig		
Resorcinol		0.40 µg/cig		1.74 µg/cig		5.42 µg/cig		
Catechol		4.0 µg/cig		10.5 µg/cig		36.4 µg/cig		
Phenol		1.60 µg/cig		2.08 µg/cig		13.8 µg/cig		
m+p Cresols		0.40 µg/cig		1.53 µg/cig		4.29 µg/cig		

o-Cresol		0.40 µg/cig		1.07 µg/cig		6.61 µg/cig		
NFDPM	S014U (AMD-1608)	1 mg/cig	T-115	0.237 mg/cig	T-212	0.593 mg/cig		
Nicotine		0.10 mg/cig		0.004 mg/cig		0.011 mg/cig	AMD-1615	4.0 mg/g (0.4%)
TPM		1 mg/cig		0.200 mg/cig		0.500 mg/cig		
**Selected Volatiles**	S094N (AMD-1673)		T-116		T-213			
1,3-Butadiene		2.02 µg/cig		0.97 µg/cig		26.4 µg/cig		
Isoprene		8.25 µg/cig		8.86 µg/cig		12.0 µg/cig		
Acrylonitrile		0.33 µg/cig		0.94 µg/cig		23.5 µg/cig		
Benzene		1.42 µg/cig		4.63 µg/cig		9.74 µg/cig		
Toluene		2.10 µg/cig		8.32 µg/cig		16.0 µg/cig		
Styrene	S020U (AMD-1614)	0.80 µg/cig	T-112	0.56 µg/cig		14.8 µg/cig		
CO	S010U (AMD-1607)		T-115	0.223 mg/cig	T-214	19.1 mg/cig		
CO_2					T-214[b]	30.0 mg/cig		
**Sugars**							AMD-1615	
Total sugars								25 mg/g (2.5%)
Reducing sugars								25 mg/g (2.5%)
**Polyphenols**							AMD-1600	
Caffeic acid								0.03 mg/g
Chlorogenic acid								1.0 mg/g
Rutin								0.5 mg/g
Scopoletin								0.05 mg/g
Total nitrogen							AMD-1647	6.5 mg/g (0.65%)
Protein nitrogen							AMD-1648	0.11% (WWB)

Table S6. Blend chemistry of experimental cigarettes

Component	Units (dwb)	K111/D111/ S111	K711/D711/ S711	HAC loading cigarettes	RTP2 Blend	CC7 Blend
Nicotine	%	2.4	2.2	2.2	2.1	2.1
Reducing sugars	%	8.8	7.2	7.2	10.2	7.8
Total sugars	%	8.9	7.3	7.3	10.9	8.2
Moisture content	%	6.8	6.8	6.8	12.6	13.2
Total nitrogen	%	2.3	2.3	2.3	1.9	3.1
Protein nitrogen	%	0.91	0.87	0.87	0.53	1.19
Caffeic acid	mg/g	0.18	0.15	0.15	0.084	0.133
Chlorogenic acid	mg/g	6.6	5.71	5.71	6.8	4.44
Rutin	mg/g	5.36	4.44	4.44	2.2	3.55
Scopoletin	mg/g	0.41	0.33	0.33	0.13	0.16
Chromium	µg/g	0.78	0.76	0.76	1.67	2.1
Nickel	µg/g	1.15	0.87	0.87	1.43	2.16
Selenium	µg/g	0.12	0.11	0.11	0.19	0.1
Arsenic	µg/g	0.11	0.11	0.11	0.14	0.2
Cadmium	µg/g	0.68	0.65	0.65	1.03	0.96
Lead	µg/g	0.36	0.34	0.34	0.32	0.66
Mercury	µg/g	<0.13	<0.13	<0.13	NA	NA
NAB	µg/g	0.02	0.02	0.02	0.018	0.044
NAT	µg/g	0.28	0.33	0.33	0.416	0.952
NNK	µg/g	0.10	0.13	0.13	0.141	0.283
NNN	µg/g	0.18	0.22	0.22	0.189	1.636
Benzo[a]pyrene	ng/g	75.6	76.1	76.1	13.8	52
